# Dual antiplatelet therapy use after non-cardioembolic ischemic stroke or transient ischemic attack: a meta-analysis of trials and cohort studies

**DOI:** 10.3389/fneur.2025.1750241

**Published:** 2026-01-12

**Authors:** Admire Hlupeni, Yusra Arooj, Adebisi Adejola, Thomas Pohlman, Ravi Donepudi

**Affiliations:** 1 Department of Internal Medicine, St Luke’s Hospital, Chesterfield, MO, United States; 2 Midlands State University, Gweru, Zimbabwe

**Keywords:** dual antiplatelet therapy (DAPT), ischemic stroke (IS), major bleeding, meta-analysis, stroke recurrence, transient ischemic attack (TIA)

## Abstract

**Background:**

Recurrent stroke burden exceeds 10% within the first year. Dual antiplatelet therapy (DAPT) is recommended for short-term secondary prevention after non-cardioembolic high-risk TIA or ischemic stroke (nc-IS), yet uncertainty persists regarding its true efficacy, optimal timing, duration, regimen, and bleeding risk.

**Aims:**

To evaluate the efficacy and safety of DAPT vs. single antiplatelet therapy (SAPT) after TIA or nc-IS and to identify clinical and study-level effect modifiers.

**Methods:**

PubMed, Embase, and Scopus were searched through October 13, 2025, for randomized trials (RTs) and cohort studies comparing DAPT and SAPT in adults (≥18 years) with TIA or nc-IS. The review was registered in PROSPERO (CRD420251017979). Recurrent stroke and major bleeding were the primary efficacy and safety outcomes of interest, respectively. Eligible studies included those comparing any DAPT vs. SAPT regimen reporting recurrent ischemic stroke or major bleeding. Two reviewers independently screened studies using predefined criteria and resolved discrepancies by consensus. Data were extracted independently by two reviewers following PRISMA guidelines. Study quality was assessed using the Cochrane RoB 2 and Newcastle–Ottawa tools. Pooled risk ratios (pRRs; 95% CIs) were calculated using random-effects models. Subgroup and meta-regression analyses explored treatment modifiers, and certainty of evidence was graded using GRADE. Data were analyzed using Stata version 18.5.

**Results:**

Twenty-seven studies (18 RTs and 9 observational; 123,136 participants) were included. DAPT was associated with significant reduction in stroke recurrence compared with SAPT (pRR 0.83; 95% CI 0.78–0.88; I^2^ = 57%). Benefit was greatest when DAPT was initiated ≤24 h-7 days and continued short-term (≤90 days), particularly when initiated with a loading dose. Efficacy was consistent across age, sex, stroke severity (including NIHSS >3–≤15), study design, and geographic setting, and extended to other DAPT regimens beyond aspirin-clopidogrel combinations. Major bleeding occurred more often with DAPT (pRR 1.29; 95% CI 1.00–1.66; I^2^ = 60%). Bleeding risk appeared slightly higher with aspirin-clopidogrel regimens and among women but was otherwise consistent across subgroups.

**Conclusion:**

Early, time-limited DAPT with a loading dose was associated with lower stroke recurrence and modest bleeding risk, with benefits extending to mild-to-moderate strokes and alternative combinations beyond the standard aspirin–clopidogrel.

**Systematic review registration:**

https://www.crd.york.ac.uk/PROSPERO, identifier CRD420251017979.

## Introduction

Recurrent ischemic stroke (IS) remains a major global health burden, with recurrence rates of 4–12% within one year and up to 20% within five years (1, 2), fuelling ongoing debate over the most effective preventive strategies. Dual antiplatelet therapy (DAPT) has emerged as a promising approach, yet its optimal use―who should receive it, when to start, for how long, and with which agents―remains uncertain.

Landmark trials such as CHANCE, POINT, and THALES showed that short-term DAPT reduces early recurrent stroke after high-risk transient ischemic attack (TIA) or minor IS (3–5), whereas SPS3 and PRoFESS found no efficacy benefit but higher bleeding and mortality risks (6, 7). Consequently, AHA/ASA guidelines currently recommend short-term DAPT only for selected patients with non-cardioembolic TIA or minor IS (8).

However, major gaps persist regarding the optimal timing of initiation, duration of therapy, and efficacy and safety of antiplatelet combinations beyond aspirin-clopidogrel (ASA-CLOP) (8). Additionally, previous meta-analyses focused mainly on randomized trials, often excluding real-world data and neglecting key modifiers such as loading dose, treatment duration, and DAPT timing (9–11).

Therefore, this meta-analysis aims to integrates all available evidence from randomized and observational studies to clarify the efficacy (recurrent stroke reduction) and safety (major bleeding risk) of DAPT vs. single antiplatelet therapy (SAPT) in non-cardioembolic TIA and IS, and to examine how these outcomes vary by DAPT timing, regimen, loading dose, duration, patient characteristics, and study design.

## Methods

### Study design

This systematic review and meta-analysis was prospectively registered with PROSPERO (CRD420251017979) (12) and conducted per PRISMA guidelines (13).

### Eligibility criteria

Population (P)

- Adults (≥18 years) with non-cardioembolic TIA or IS of NIH Stroke Scale (NIHSS) ≤ 15. Studies limited to a single etiologic subtype such as lacunar or large-artery stroke, using surrogate non-clinical outcomes, or involving thrombolysis, thrombectomy, or anticoagulation were excluded.

Intervention (I)

- DAPT, defined as the concurrent use of two antiplatelet agents. Ticlopidine regimens were excluded due to limited contemporary use and risk of serious hematologic toxicity.

Comparator (C)

- SAPT

Outcomes (O)

- The primary outcomes were recurrent stroke—either as a standalone endpoint or as part of a composite vascular outcome—and major bleeding, as defined in each study.

Study Design (S)

- Eligible studies were randomized trials (RTs) or cohort studies comparing DAPT and SAPT. Secondary or exploratory post-hoc studies were excluded.

### Search strategy

We systematically searched PubMed, Embase, and Scopus from inception through October 13, 2025 using a search strategy described in the protocol (12). Searches were limited to peer-reviewed, published articles in English. Grey literature, conference abstracts, animal studies, and unpublished data were excluded.

### Study selection

Articles were managed in Rayyan which automatically flagged potential duplicates. One investigator then manually decided which copies to retain or delete. Two reviewers independently screened titles and abstracts for relevance, and full-text articles of potentially eligible studies were retrieved and assessed for inclusion. Discrepancies were resolved through discussion or by consulting a third reviewer.

### Data extraction & methodological quality assessment

Two reviewers independently extracted study and patient characteristics, treatment variables (regimen, loading dose, timing, duration), and outcomes using a standardized form. Disagreements were resolved by consensus. Methodological quality was assessed at the study level using the Cochrane RoB 2 tool for RTs and the Newcastle–Ottawa Scale (NOS) for cohort studies. These appraisals informed the risk-of-bias domain within the GRADE framework used to rate the overall certainty of evidence.

### Data synthesis

Pooled risk ratios (pRRs) with corresponding 95% confidence intervals (CIs) were calculated using random-effects models based on the DerSimonian and Laird method, to account for expected clinical and methodological heterogeneity across studies. Statistical heterogeneity was quantified using the I^2^ statistic.

Pre-specified subgroup analyses were conducted to explore potential effect modifiers and sources of heterogeneity. Age and sex were included given their established influence on stroke outcomes. Following convention in vascular and stroke literature, age 65 years was used as a reference threshold to distinguish studies enrolling predominantly older versus younger populations, while the proportion of male participants described sex distribution. Both variables, mean participant age and proportion of male participants, were analyzed as continuous moderators using random-effects meta-regression.

To align with AHA/ASA secondary stroke prevention guidelines (8) and prior clinical trials (3, 4), IS was dichotomized as minor (NIHSS ≤3) or mild-to-moderate (NIHSS >3 to ≤15). Studies lacking NIHSS data were excluded from this subgroup analysis. For studies including patients with TIAs but not specifying the ABCD^2^ score, populations were assumed to represent high-risk TIAs, consistent with the inclusion criteria in major DAPT trials.

Similarly, time from symptom onset to DAPT initiation was classified as early (≤24 h), delayed (≤7 days), and late (>7 days), in line with the AHA/ASA guideline recommendations, which advise initiating DAPT as early as possible—ideally within 12–24 h and at least within 7 days of symptom onset—to reduce recurrent IS risk (8).

Dual antiplatelet therapy regimens were categorized as ASA-CLOP or non-ASA-CLOP combinations. The latter included regimens such as aspirin plus ticagrelor, aspirin plus dipyridamole, aspirin plus cilostazol, or cilostazol plus clopidogrel. Regimens containing ticlopidine were excluded. Additional subgroup analyses examined the influence of use of an initial loading dose, study design (RCT vs. observational study), and geographic setting (single-country vs. multicountry studies).

Small-study effects was assessed by visual inspection of funnel plots and using Egger’s regression test. Where evidence of small-study effects was observed, trim-and-fill analysis was applied to estimate potentially missing studies and adjust pooled effect estimates accordingly. All analyses were conducted using Stata version 18.5 (StataCorp, College Station, TX).

### Certainty of evidence

Certainty of evidence was evaluated using the GRADE framework, considering study design, risk of bias, inconsistency, indirectness, imprecision, and potential publication bias.

## Results

### Study selection

**Figure 1:**
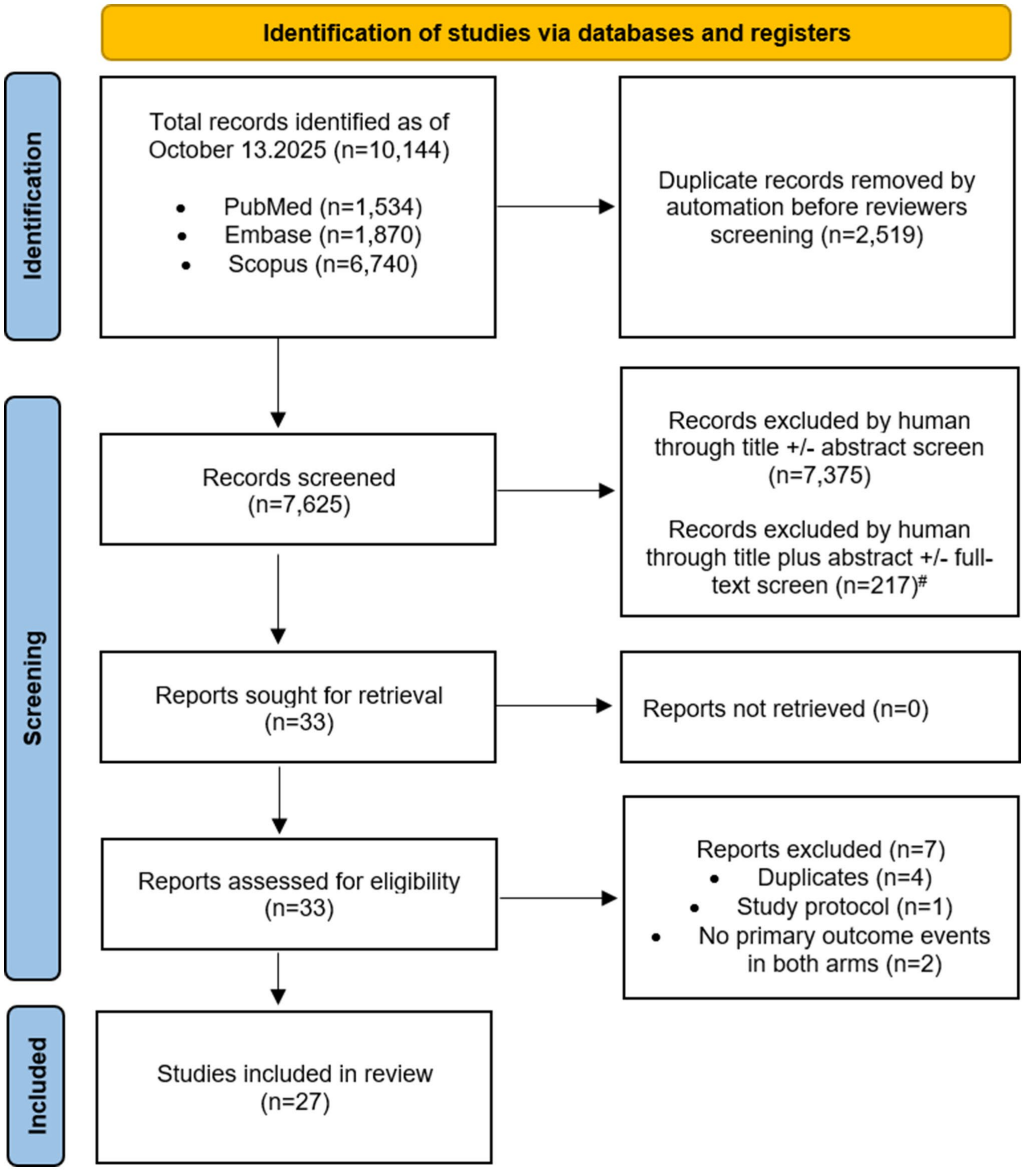
PRISMA flow diagram of study selection for the meta-analysis. ^#^ Wrong study design (including reviews, case report or series, pooled analyses, subgroup analyses) *n* = 130; Wrong intervention or comparator (including wrong study drug) *n* = 35; Not relevant to research question *n* = 32; Wrong outcome of interest (including surrogate and non-clinical endpoints) *n* = 27; Wrong study population (including studies that included only specific subtypes of strokes, asymptomatic carotid disease, hemorrhagic strokes) *n* = 26; Wrong publication type (including editorials, letters, comments, protocols) *n* = 4; Unavailable full text *n* = 3; and overlap *n* = 1. Because several studies met more than one exclusion criterion, the sum of reasons for exclusion exceeds the total number of excluded articles (*n* = 217).

Of 10,144 records identified, 27 studies (18 randomized, 9 observational) met inclusion criteria (Figure 1). Baseline stroke severity (NIHSS or equivalent descriptors), TIA risk classification, and definitions of major bleeding outcomes are summarized in Supplementary Table S1. High-profile trials such as SPS3 (6), COMPRESS (14), and CARESS (15) were excluded for focusing on distinct stroke subtypes or surrogate outcomes. Key exclusions and rationales appear in Supplementary Table S2.

### Quality assessment for included studies

Most RTs were at low risk of bias across all domains, though some had “some concerns” in one or more domains (such as deviations from intended interventions, and inadequate and selective study results). According to RoB 2 guidelines, a single domain with “some concerns” resulted in an overall judgment of “some concerns” for that trial (Supplementary Table S3). Observational studies were generally of moderate to high quality, with total NOS scores ranging from 7 to 9 (Supplementary Table S4).

### Efficacy outcome

**Figure 2:**
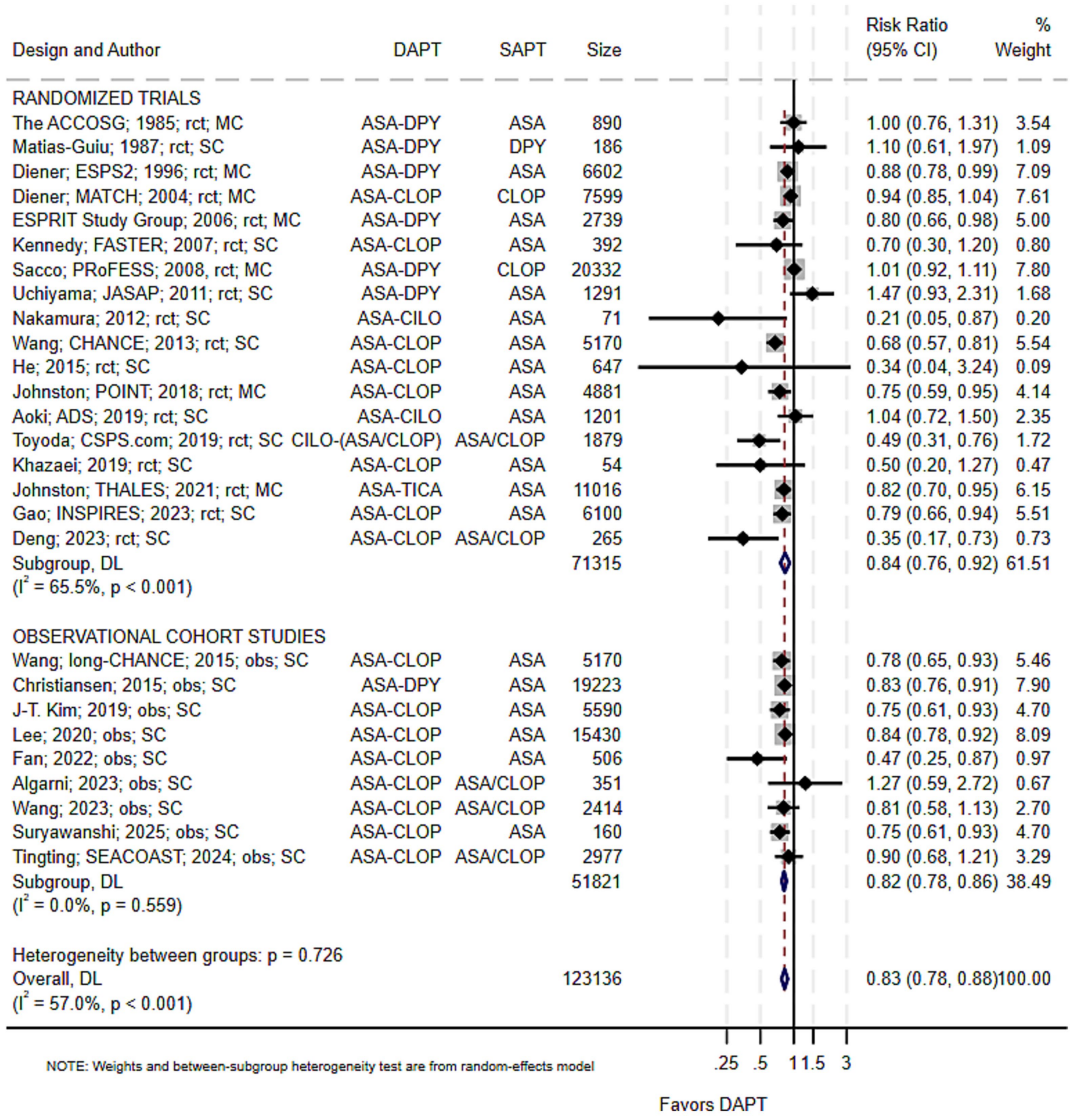
Pooled risk of recurrent stroke with dual versus single antiplatelet therapy. ASA, aspirin; CLOP, clopidogrel; CILO, cilostazol; DAPT, dual antiplatelet therapy; DPY, dipyridamole; MC, multicountry; obs, observational cohort; RCT, randomized trial; SC, single country; SAPT, single antiplatelet therapy. Study details are presented as the first author; ± study acronym; publication year; study design; study setting. *ASA–CLOP* denotes the aspirin–clopidogrel combination, and *ASA/CLOP* indicates either aspirin or clopidogrel monotherapy. The same notation applies to other dual and single antiplatelet combinations.

Twenty-seven studies (123,136 participants), including 18 RCTs and 9 cohort studies, were included (Table 1). Overall, DAPT was associated with a significantly lower risk of recurrent stroke compared with SAPT (pRR = 0.83; 95% CI 0.78–0.88; *p* < 0.001; I^2^ = 57.0%) (Figure 2). Funnel plot asymmetry and Egger’s test (*p* = 0.05) suggested potential small-study effects. However, after adjustment for three imputed studies using the trim-and-fill method (Supplementary Figure S1), the pooled estimate was minimally attenuated (pRR = 0.84; 95% CI 0.79–0.89), supporting the robustness of the primary findings.

**Table 1:** Summary of the studies included in the meta-analysis of dual versus single antiplatelet therapy after non-cardioembolic transient ischemic attack and ischemic stroke.

Author	Design	Setting	Size	Efficacy outcome	Bleeding outcome	DAPT	SAPT	TIA%^c^	IS %^c^	Male% ^c^
The American-Canadian Co-Operative Study Group (1985); Persantine-Aspirin Trial (19)	RT	USA & Canada	890	Composite (stroke, retinal infarction, or death from any cause).	–	ASA + DPY	ASA	100	0	67.0
J. Matias-Guiu (1987) (20)	RT	Spain	186	Reversible ischemic attacks and completed strokes.	–	ASA + DPY	DPY	81.2	18.8	76.3
HC. Diener (1996); ESPS2^a^ (21)	RT	Europe	6,602	Composite (stroke and/or death).	Moderate & severe or fatal bleeding (any site).	ASA + DPY	ASA/DPY	23.7	76.3	58.0
HC. Diener (2004); MATCH^a^ (22)	RT	MC	7,599	Composite (IS, MI, vascular death or rehospitalization for acute ischemic event)	Life threatening and major bleeding	ASA + CLOP	CLOP	21.0	79.0	63.0
The ESPRIT Study Group (2006)^a^ (23)	RT	MC	2,739	Composite (Death from all vascular causes, non-fatal stroke, non-fatal MI, or major bleeding complication).	Moderate & severe or fatal bleeding (any site).	ASA + DPY	ASA	33.6	66.4	65.3
J. Kennedy (2007); FASTER (24)	RT	Canada	392	Total strokes (Ischemic and hemorrhagic)	–	ASA + CLOP	ASA	-	-	52.8
RL. Sacco (2008); PRoFESS^a^ (7)	RT	MC	20,332	First recurrent stroke of any type	Major bleeding^b^	ASA + DPY	CLOP	0	100	64
S. Uchiyama (2011); JASAP^a^ (25)	RT	Japan	1,291	IS (fatal or non-fatal)	Major bleeding^b^	ASA + DPY	ASA	0	100	71.5
T. Nakamura (2012) (26)	RT	Japan	71	Neurological deterioration or stroke recurrence	–	ASA + CILO	ASA	0	100	73.7
Y. Wang (2013); CHANCE^a^ (3)	RT	China	5,170	Stroke (ischemic or hemorrhagic)	Moderate-to-severe bleeding^b^	ASA + CLOP	ASA	27.9	72.1	66.2
F. He (2015) (27)	RT	China	647	Composite (Neurological deterioration or stroke)	–	ASA + CLOP	ASA	5.9	94.1	56.9
Y. Wang (2015)^a^ (28)	O	China	5,170	New stroke (ischemic or hemorrhagic)	Moderate-to-severe bleeding^b^	ASA + CLOP	ASA	27.9	72.1	66.2
CB. Christiansen (2015) (29)	O	Denmark	19,223	IS	–	ASA + DPY	ASA	0	100	-
SC. Johnston (2018); POINT^a^ (4)	RT	MC	4,881	Composite (IS, MI, or death from ischemic vascular causes)	Major hemorrhage^b^	ASA + CLOP	ASA	43.2	56.8	55.0
J. Aoki (2019)^a^ (30)	RT	Japan	1,201	Composite (neurological deterioration, symptomatic stroke or TIA)	Intracerebral & subarachnoid hemorrhage	ASA + CILO	ASA then CILO	0	100	66.0
K. Toyoda (2019); CSPS. com^a^ (31)	RT	Japan	1879	Symptomatic IS	Severe or life- threatening bleeding	ASA/ CLOP + CILO	ASA	0	100	70.3
M. Khazaei (2019)^a^ (32)	RT	Iran	54	Secondary TIA	Gastrointestinal hemorrhage	ASA + CLOP	ASA	66.7	33.3	63.0
J-T. Kim (2019) (33)	O	Korea	5,590	Composite (ischemic and hemorrhagic strokes, MI & vascular death)	–	ASA + CLOP	ASA	6.9	93.1	62.6
H-L. Lee (2020) (34)	O	Korea	15,430	Composite (hemorrhagic or ischemic stroke, MI & all-cause mortality)	–	ASA + CLOP	ASA	0	100	62.0
SC. Johnston (2021); THALES-reanalysis^a 5^	RT	MC	11,016	Composite (IS or non-hemorrhagic death).	Major bleeding (composite of ICH and fatal bleedings)	ASA + TICA	ASA	9.4	90.6	61.2
H. Fan (2022)^a^ (35)	O	China	506	Composite (IS, TIA, MI & moderate-to-severe bleeding).	Moderate-to-severe bleeding^b^	ASA + CLOP.	ASA.	0	100	72.5
Y. Gao (2023); INSPIRES^a^ (36)	RT	China	6,100	New stroke (ischemic or hemorrhagic)	Moderate-to-severe bleeding^b^	ASA + CLOP	ASA	13.1	86.9	64.2
RA. Algarni (2023) (37)	O	Saudi Arabia	351	Composite (IS, rehospitalization & all-cause mortality)	–	ASA + CLOP	ASA/ CLOP	0	100	65.2
L. Wang (2023) (38)	O	China	2,414	Recurrent stroke	–	ASA + CLOP	ASA/ CLOP	0	100	68.0
T. Deng (2023) (39)	RT	China	265	Recurrent IS	–	ASA + CLOP	ASA/ CLOP	0	100	72.1
T. Liu (2024); SEACOAST^a^ (40)	O	China	2,977	Composite (IS, TIA, symptomatic intracerebral hemorrhage, MI or angina attacks & vascular death)	Severe bleeding^b^	ASA + CLOP	ASA/ CLOP	0	100	73.3
VR. Suryawanshi (2025) (41)	O	India	160	Early neurological deterioration & new stroke or TIA.	–	ASA + CLOP	ASA	12.0	88.0	62.0

ASA, aspirin; CILO, cilostazol; CLOP, clopidogrel; DAPT, dual antiplatelet therapy; O, observational study; RT, randomized trial; SAPT, single antiplatelet therapy; TICA, ticagrelor; IS, ischemic stroke; MI, myocardial infarction; TIA, transient ischemic attack; MC, multicountry; ICH, intracranial hemorrhage. *ASA/CLOP* indicates either aspirin or clopidogrel monotherapy.

^a^Included in the safety outcome analysis; ^b^defined according to GUSTO criteria; ^c^denotes the percentage of TIA, IS, or male participants in the study population.

#### Subgroup analyses

**Figure 3:**
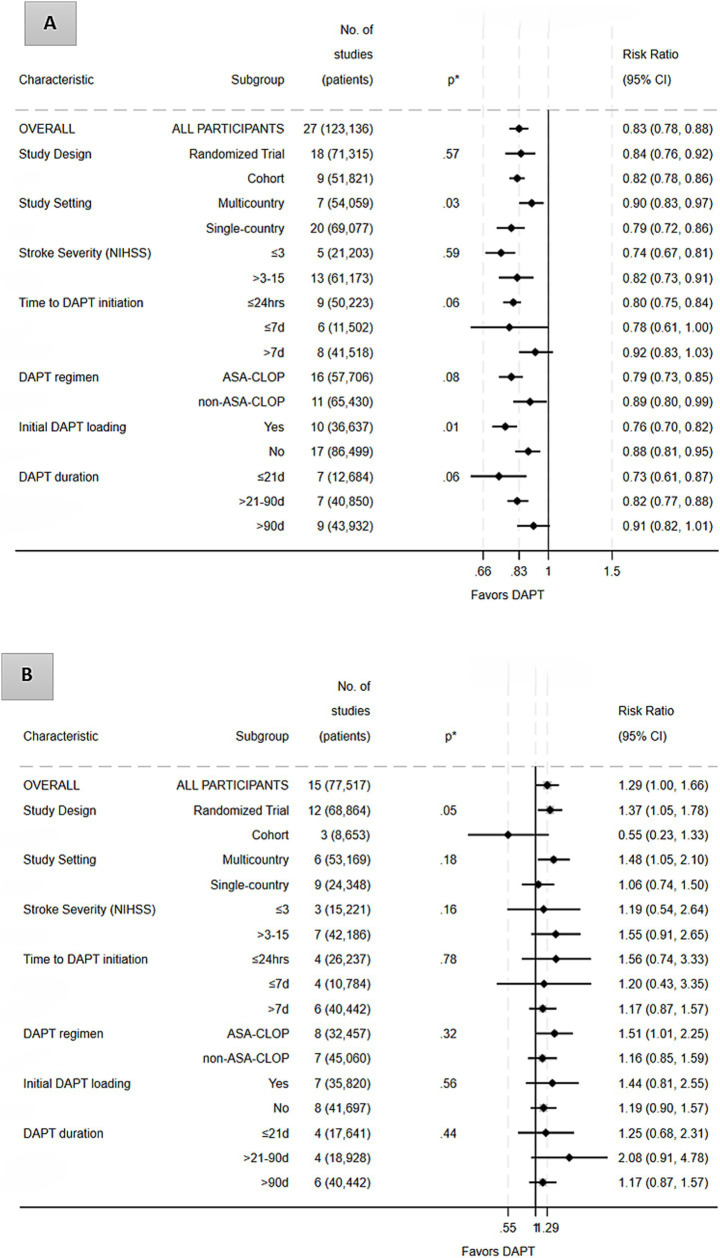
Subgroup analyses for the efficacy outcome (recurrent stroke prevention; Panel **A**) and the safety outcome (major bleeding; Panel **B**). ASA, aspirin; CLOP, clopidogrel; DAPT, dual antiplatelet therapy; M, multi-country; non-ASA–CLOP, dual antiplatelet combinations other than aspirin–clopidogrel (e.g., aspirin–ticagrelor, aspirin–cilostazol, aspirin–dipyridamole, cilostazol–clopidogrel); NIHSS, National Institutes of Health Stroke Scale; d-days; hrs-hours. p*-indicates the *p* value for between-subgroup difference (test for interaction).

Subgroup analyses showed that the benefit of DAPT for preventing recurrent stroke was broadly consistent across study designs, treatment regimens, and patient characteristics (Figure 3A; Supplementary Figures S2–S7).

Analyses based on DAPT loading strategy revealed a significant difference in treatment effect between studies that used a loading dose and those that did not (*p* = 0.01). While both strategies were associated with reduced stroke recurrence, the benefit was more pronounced with loading (pRR 0.76; 95% CI 0.70–0.82) than without loading (pRR 0.88; 95% CI 0.81–0.95), suggesting that initial intensification of platelet inhibition confers additional early benefit.

The efficacy of DAPT was strongly time-dependent. The greatest relative risk reduction in recurrent stroke was observed when DAPT was administered for ≤21 days (RR 0.73; 95% CI 0.61–0.87), with a sustained but smaller benefit when continued for up to 90 days (RR 0.82; 95% CI 0.77–0.88). Beyond 90 days, the treatment effect was attenuated and no longer statistically significant (RR 0.91; 95% CI 0.82–1.01), indicating diminishing returns with prolonged therapy.

A similar time-dependent gradient was observed with respect to treatment initiation. The most pronounced benefit occurred when DAPT was started within 24 h of the index event (RR 0.80; 95% CI 0.75–0.84), with a comparable effect when initiated within ≤7 days (RR 0.78; 95% CI 0.61–1.00). In contrast, delayed initiation beyond 7 days was associated with a smaller and statistically nonsignificant reduction in recurrent stroke (RR 0.92; 95% CI 0.83–1.03). The subgroup comparison approached statistical significance (*p* = 0.056), suggesting that earlier initiation of DAPT provides greater protection during the early high-risk period after stroke or TIA.

When stratified by study setting, multicountry studies reported a slightly higher pooled relative risk (pRR = 0.90; 95% CI 0.83–0.97) compared with single-country studies (pRR = 0.79; 95% CI 0.72–0.86), with a statistically significant between-group difference (*p* = 0.03).

In contrast, no significant differences in treatment effect were observed across analyses stratified by DAPT regimen (ASA-CLOP vs. non-ASA-CLOP combinations, including aspirin-dipyridamole, aspirin-cilostazol, aspirin-ticagrelor, and cilostazol-clopidogrel; *p* = 0.08), baseline stroke severity (minor stroke, NIHSS ≤ 3 vs. mild-to-moderate stroke, NIHSS > 3–15; *p* = 0.16), or study design (RTs vs. observational cohorts; *p* = 0.73).

Similarly, meta-regression analyses showed that neither mean age (*β* = 0.01; 95% CI –0.02–0.03; *p* = 0.57) nor the proportion of male participants (*β* = −0.0004; 95% CI –0.02–0.02; *p* = 0.96) significantly influenced the treatment effect.

### Safety outcome

**Figure 4:**
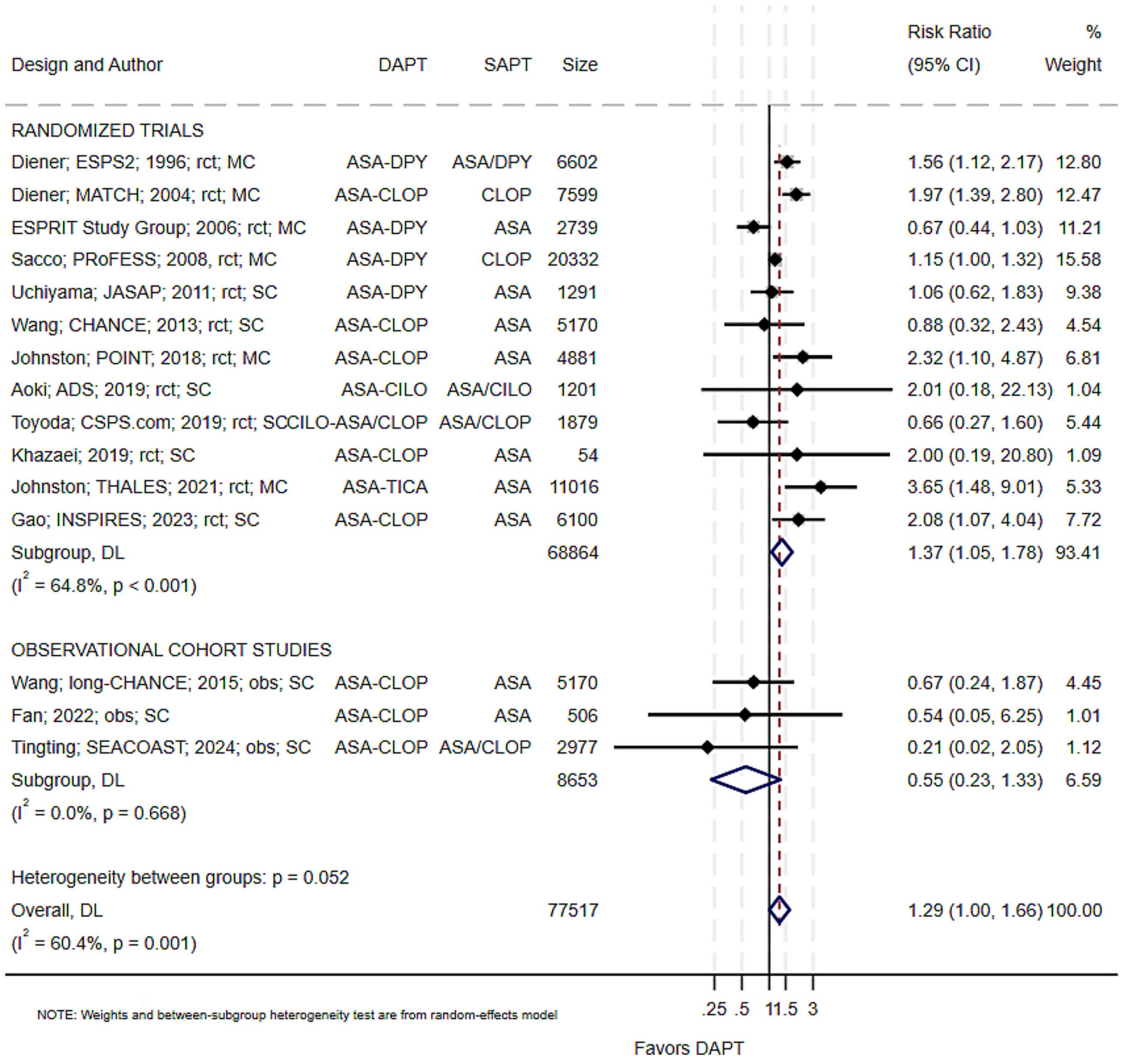
Pooled risk of major bleeding with dual versus single antiplatelet therapy. ASA, aspirin; CLOP, clopidogrel; CILO, cilostazol; DAPT, dual antiplatelet therapy; DPY, dipyridamole; MC, multicountry; obs, observational cohort; RCT, randomized trial; SC, single country; SAPT, single antiplatelet therapy. Study details are presented as the first author; ± study acronym; publication year; study design; study setting. *ASA–CLOP* denotes the aspirin–clopidogrel combination, and *ASA/CLOP* indicates either aspirin or clopidogrel monotherapy. The same notation applies to other dual and single antiplatelet combinations.

Of the 27 included studies, 15 (12 RCTs and 3 observational studies; 77,517 participants) reported major bleeding events in both arms and were included in the safety analysis (Table 1). Overall, DAPT was associated with a significantly increased risk of major bleeding compared with SAPT (pRR 1.29; 95% CI 1.00–1.66; *p* = 0.001; I^2^ = 60.4%) (Figure 4). The lower confidence boundary at unity indicates that, although statistically significant, the increase in bleeding risk was modest and borderline in magnitude. Funnel-plot inspection and Egger’s test (*p* = 0.89) did not demonstrate evidence of small-study effects.

#### Subgroup analyses

Across subgroup analyses the pattern of bleeding risk was directionally consistent but varied slightly by treatment and study characteristics (Figure 3B; Supplementary Figures S8–S13).

When stratified by study design, RCTs showed a significant increase in major bleeding risk (pRR 1.37; 95% CI 1.05–1.78; I^2^ = 64.8%), whereas observational cohorts did not (pRR 0.55; 95% CI 0.23–1.33; I^2^ = 0%). The between-group comparison approached significance (*p* = 0.05), suggesting a potential design-related difference that may reflect more rigorous event adjudication and reporting in trials than in observational studies.

Although the difference did not reach statistical significance (*p* = 0.32), there was a numerical trend toward higher bleeding risk with ASA-CLOP combination (pRR 1.51; 95% CI 1.01–2.25) compared with non-ASA-CLOP regimens (pRR 1.16; 95% CI 0.85–1.59).

Bleeding risk did not differ significantly between DAPT with and without a loading dose (*p* = 0.56), or across short- (≤21 days), intermediate- (21–90 days), and long-term (>90 days) DAPT treatment durations (*p* = 0.44). Likewise, no significant differences were observed when stratified by baseline stroke severity (NIHSS ≤3 vs. > 3; *p* = 0.59), study setting (multicountry vs. single-country; *p* = 0.18), or time to DAPT initiation (≤24 h, ≤7 days, >7 days; *p* = 0.78).

In meta-regression analyses, the proportion of male participants was inversely correlated with bleeding risk (*β* = −0.07; 95% CI –0.13 to −0.01; *p* = 0.02), suggesting that female participants may be relatively more susceptible to DAPT-related bleeding. Conversely, mean age was not significantly associated with bleeding risk (*β* = 0.09; 95% CI –0.07–0.25; *p* = 0.26), indicating that even in studies enrolling participants aged >65 years, older adults did not experience disproportionate bleeding with DAPT (Supplementary Figure S14).

### Certainty of evidence

Using the GRADE framework, the overall certainty of evidence was rated as high for the efficacy outcome (stroke recurrence) and moderate for the safety outcome (major bleeding). This grading reflects that most included trials were of high methodological quality with consistent direction of effect, though moderate heterogeneity and minor risk-of-bias concerns were present for bleeding outcomes. Collectively, the certainty of evidence supports a robust and reliable association between DAPT and reduced stroke recurrence, with a modest and well-characterized bleeding risk (Supplementary Table S5).

## Discussion

This comprehensive meta-analysis of 27 studies involving over 120,000 participants demonstrates that DAPT is associated with a significant reduction in recurrent ischemic stroke compared with SAPT (pRR 0.83; 95% CI 0.78–0.88), with only a modest increase in major bleeding (pRR 1.29; 95% CI 1.00–1.66). These findings reaffirm the central role of DAPT in early secondary stroke prevention and, together with subgroup analyses, refine its optimal timing, duration, regimen, and patient selection.

### Timing, duration, and intensity of DAPT

The timing of initiation emerged as a major determinant of efficacy. DAPT was associated with the greatest protection when started within 24 h of symptom onset, with sustained benefit when begun within 7 days. Beyond this window, efficacy declined, highlighting the importance of early initiation during the hyperacute phase, when recurrent stroke risk is highest.

Treatment duration also influenced benefit. The largest relative risk reduction occurred within the first 21 days, remained significant through 90 days, and declined thereafter. Interestingly, prolonged DAPT therapy beyond 90 days was not associated with increased bleeding risk, suggesting that the loss of benefit—not excess harm—limits its usefulness over time. This supports the time-limited DAPT approach recommended by current guidelines (8).

A loading-dose strategy was further associated with enhanced efficacy, underscoring the importance of achieving rapid platelet inhibition in the early phase.

Collectively, these findings show that both the timing and intensity of platelet inhibition, not only agent choice, are central to maximizing benefit.

### Stroke severity and regimen-specific effects

Efficacy was consistent across baseline stroke severities. Patients with mild-to-moderate ischemic strokes (NIHSS >3–15) benefited similarly to those with minor strokes (NIHSS ≤3), suggesting that DAPT can be safely and effectively extended to a broader spectrum of non-cardioembolic events with appropriate monitoring.

Similarly, comparable efficacy between ASA-CLOP and non-ASA-CLOP combinations (e.g., aspirin-dipyridamole, aspirin-cilostazol, cilostazol-clopidogrel) suggests that alternative dual regimens may be suitable for patients intolerant to standard therapy or at higher bleeding risk. This broadens the therapeutic landscape and supports individualized regimen selection guided by tolerance, comorbidity, and drug access.

### Safety and bleeding risk

While DAPT was modestly associated with increased major bleeding risk, the absolute excess risk was small relative to the stroke prevention benefit. Bleeding estimates differed slightly by design. Randomized trials showed a stronger signal than observational cohorts, possibly reflecting more systematic event adjudication.

A numerical trend toward higher bleeding risk with ASA-CLOP compared with other combinations suggests that additive COX-1 and P2Y12 inhibition may confer greater hemorrhagic potential. Meta-regression identified no association between age and bleeding, indicating that older adults can be treated safely with DAPT with close supervision. However, a significant inverse correlation with male proportion (*β* = −0.07; *p* = 0.01) indicates relatively higher bleeding susceptibility in women. This sex-specific signal, also noted in cardiovascular DAPT studies (16–18), may reflect biological differences in platelet response and drug metabolism. Collectively, these results call for heightened vigilance in women and in patients receiving ASA-CLOP dual therapy, where bleeding risk may be marginally higher.

Overall, this metanalysis emphasize that bleeding risk with DAPT is modest, predictable, and largely outweighed by its benefit in appropriately selected patients.

## Study limitations

Several limitations merit acknowledgment. First, inclusion was limited to published, English-language studies, introducing potential publication bias, although no major small-study effects were detected. Second, moderate heterogeneity—particularly in bleeding outcomes—likely reflects methodological differences, including variations in eligibility criteria, endpoint definitions, and follow-up duration. Third, combining RTs and observational studies improves generalizability but introduces variability in study quality and event ascertainment.

Finally, analyses were based on study-level data, precluding patient-level adjustment for confounders such as frailty, renal function, or concomitant therapies.

Despite these limitations, the large sample size, comprehensive sensitivity analyses, and consistency across subgroups strengthen the validity and clinical relevance of these conclusions.

## Clinical implications

This analysis suggests that early, short-term DAPT—initiated within 24 h-to-7 days and continued between 21 and 90 days with an initial loading dose—offers the greatest net clinical benefit for secondary stroke prevention. The absence of excess bleeding beyond 90 days suggests that the optimal treatment window is defined by waning efficacy rather than rising harm.

Clinicians should also note that DAPT appears safe in older adults and may be extended to mild-to-moderate non-cardioembolic IS when bleeding risk is acceptable. Female patients and those on ASA-CLOP regimens may require closer monitoring or consideration of alternative dual combinations such as aspirin–cilostazol or aspirin-dipyridamole. Ultimately, DAPT decisions should be individualized, balancing early ischemic protection with patient-specific bleeding risk.

## Future directions

Future research should focus on refining bleeding risk prediction tools, integrating sex-specific and biomarker-driven models, and clarifying optimal DAPT duration across diverse clinical settings. Collaborative individual-participant meta-analyses could define patient-level modifiers of benefit and harm more precisely.

Equally important, methodological heterogeneity across existing studies, especially differences in eligibility criteria, timing of outcome assessment, and endpoint definitions, complicates comparisons and pooling of aggregated effect estimates, and this may obscure true treatment effects. Harmonizing these definitions under the guidance of major organizations (AHA/ASA, ESO, and global cardiovascular consortia) may enhance trial comparability, reduce heterogeneity, and strengthen the overall evidence base for DAPT use in secondary stroke prevention.

## Conclusion

This meta-analysis reaffirms DAPT as an effective strategy for reducing recurrent stroke after TIA or ischemic stroke, particularly when initiated early, given with a loading dose, and maintained short-term. Although bleeding risk increases modestly, the net clinical benefit remains favorable. These results support current guideline-endorsed use of short-term DAPT and highlight the importance of individualized therapy balancing efficacy and safety in real-world practice.

## Data Availability

The original contributions presented in the study are included in the article/Supplementary material, further inquiries can be directed to the corresponding author.
