# Supplementary material for: Dual antiplatelet therapy use after non-cardioembolic ischemic stroke or transient ischemic attack: a meta-analysis of trials and cohort studies
Source: Front Neurol. 2026 Jan 12;16:1750241. doi: 10.3389/fneur.2025.1750241 (PMC12869719; doi:10.3389/fneur.2025.1750241)
Supplement: Supplementary file 1 [file Data_Sheet_1.pdf]

SUPPLEMENTARY DATA

Supplementary Table S1. Baseline Stroke Severity, TIA Risk Scores, and Study Definitions of Major Bleeding Across Included Studies

Author	Primary Efficacy Outcome	Primary Safety Outcome	Mean/Median age (years)	Eligibility age (years)	Male (%)	TIA (%)	ABCD² score	Ischemic Stroke (%)	NIHSS	Follow duration
The ACCSG; Persantine Aspirin Trial; 1985	Stroke, retinal infarction, or death from any cause.	-	63	<80	67.00	100.0	not mentioned	0	Not applicable	61 months (median 25 months)
J. Matias-Guiu; 1987	Recurrence of reversible ischemic attacks and completed strokes	-	55	<65	76.30	81.2 (included TIAs and RIND)	not mentioned	18.8	not mentioned	3- 40 months
H-C. Diener; ESPS2; 1996	Composite (stroke, death, stroke and/or death).	Moderate; and severe or fatal bleeding (any site). No specific definition given	66.7	>18	58.00	23.7	not mentioned	76.3	mRS 5 and less. NIHSS not mentioned	2 years
HC. Diener, et al; MATCH; 2004	Composite (First occurrence: ischemic stroke, MI, vascular death or rehospitalization for acute	Life-threatening bleeding (defined as any fatal bleeding event; a drop in hemoglobin of _50 g/L; significant	66.3	40 and above	63.00	21.0	not mentioned	79.0	mRS up to 5. NIHSS not mentioned	18 months

	ischemic event)	hypotension with need for inotropes [hemorrhagic shock]; symptomatic intracranial hemorrhage, or transfusion of _4 units of red-blood cells or equivalent amount of whole blood) and major bleeding (defined as significantly disabling [with persistent sequelae]; intraocular bleeding leading to significant loss of vision; or transfusion of _3 units of red-blood cells or equivalent amount of whole blood).								
The ESPRIT Study Group;	Composite (death from all vascular	Major bleeding complications (intracranial	63	Not specified	65.30	33.6	Not mentioned	66.4	mRS 3 and less. NIHSS not	5 years, mean 3.5 years

ESPRIT; 2006	causes, non-fatal stroke, non-fatal myocardial infarction, or major bleeding complication, whichever happened first).	bleeding, any fatal bleeding, or any bleeding requiring hospital admission.)							mentioned	
J. Kennedy; FASTER; 2007	Total strokes (ischemic and hemorrhagic) within 90 days	-	68.1	40 and above	52.80	not mentioned	not mentioned	not mentioned	NIHSS 3 or less	90 days
R L. Sacco; PRoFESS; 2008	First recurrent stroke of any type	Major hemorrhage events (life-threatening or non–life-threatening) was defined as a hemorrhagic event that resulted in clinically significant disability, symptomatic intracranial hemorrhage, intraocular bleeding causing loss of	66.1	50 or older	64.00	0.0	Not applicable	100%	Approximately 14.3% of the study patients had >5 NIHSS strokes.	1.5-4.4 years, mean 2.5 years

		vision, the need for a transfusion of 2 or more units of red cells or the equivalent amount of whole blood, or the need for hospitalization. Life-threatening hemorrhagic events were defined as those that were fatal or that required use of intravenous inotropic medication to maintain blood pressure, surgical intervention, or transfusion of 4 or more units of red cells or the equivalent amount of whole blood. Non–life-threatening hemorrhagic events								

		were defined as those classified as major hemorrhagic events but not as life-threatening								
S. Uchiyama; JASAP; 2010	Recurrent ischemic stroke (fatal or non-fatal)	Major bleeding events Defined as at least 1 of the following: fatal hemorrhage; retroperitoneal hemorrhage, intracranial hemorrhage, intraocular hemorrhage (objective finding and subjective symptom leading to bleeding) or spinal/intraspinal hemorrhages (confirmed by objective findings); bleedings requiring surgery;	66.1	50 or older	71.50	0.0	Not applicable	100%	mRS less than 4. NIHSS not mentioned	704 days

		clinically obvious bleeding requiring ≥4.5 units of blood transfusion or accompanied by a ≥2 g/dl decrease in hemoglobin level.								
T. Nakamura; 2012	Composite (Neurological deterioration or stroke recurrence of increased NIHSS scores by 1 or more within 14 days).	-	Mean (SD): SAPT 67 +/- 10, DAPT 66 +/- 12.	Not specified	73.70	0.0	Not applicable	100	NIHSS less than 8. mRS less than 4.	6 months (outcomes compared at 14 days)
Y. Wang; CHANCE; 2013	Stroke (ischemic or hemorrhagic) during 90 days of follow-up	Moderate-to-severe bleeding as per GUSTO guidelines	62	40 or older	66.20	27.9	4 and above (median 4)	72.1	NIHSS less than 4	90 days
F. He; 2014	Composite (neurological deterioration, recurrent stroke, stroke in a patient with a TIA	-	62.2	40 and older	56.90	5.9	not mentioned	94.10	NIHSS 7 and less.	14 days

	within 14 days)									
Y. Wang; CHANCE- Long term follow-up; 2015	New stroke event (ischemic or hemorrhagic) during 1 year of follow-up.	Moderate-to-severe bleeding defined according to the Global Utilization of Streptokinase and Tissue Plasminogen Activator for Occluded Coronary Arteries (GUSTO) definition.	62.5	40 and older	66.20	27.9	4 and above (median 4)	72.1	NIHSS 3 or less	1 year
CB. Christiansen; 2015	1-year risk of recurrent ischemic stroke	-	Median (IQR): SAPT 75.3 (64.4-83.7), DAPT 70.7 (61.5-79.6)	Not specified	not specified for the entire cohort	0.0	Not applicable	100	Not mentioned	Median 335 days (IQR 335-335)
SC. Johnston; POINT; 2018	Composite (ischemic stroke, myocardial infarction, or death from ischemic vascular causes (major	Major hemorrhage defined as symptomatic intracranial hemorrhage, intraocular bleeding causing vision loss,	65	18 and above	55.00	43.2	4 and above (median 5)	56.8	NIHSS 3 or less	90 days (with a window of ±14 days)

	ischemic events)).	transfusion of 2 or more units of red cells or an equivalent amount of whole blood, hospitalization or prolongation of an existing hospitalization, or death due to hemorrhage								
J. Aoki; ADS; 2019	Any one of the following occurring within 14 days of onset: neurological deterioration, symptomatic stroke recurrence, and TIA.	Intracerebral and subarachnoid hemorrhage assessed at 14 days	69	18 and above	66.00	0.0	Not applicable	100	NIHSS 20 and less (median 2, IQR 1-4). mRS 0-2.	3 months, but outcomes assessed at day 14.
K. Toyoda; CSPS.com; 2019	First recurrence of symptomatic ischemic stroke	Severe or life-threatening bleeding	69.6	Between 20 and 85	70.30	0.0	Not applicable	100	90% had mRS 0-1. NIHSS not mentioned	upto 3.5 years (median 1.4 years)
	secondary TIA	Gastrointestinal hemorrhage	67.3	Not specified	63.00	66.7	Not mentioned	33.3	NIH 5 or less	3 months

J-T. Kim; 2019	Composite (ischemic and hemorrhagic), MI, and vascular death by 3 months.	-	64	40 and above	62.60	6.9	Not mentioned	93.10	NIHSS 3 or less (median 1)	median 93 days, IQR 93-93
H-L. Lee, et al; 2020	3-month composite of stroke (either hemorrhagic or ischemic), MI, & all-cause mortality.	-	66	Not specified	62.00	0.0	Not applicable	100	NIHSS 10 and less (median 3 (IQR 1-5))	mean (SD) 89.7 +/- 22.4 days
SC. Johnston; THALES - reanalysis; 2021	Composite (ischemic stroke or non-hemorrhagic death)	Major bleeding (composite of ICH and fatal bleedings)	65	40 and above	61.20	9.4	87.3% had a score of 6 and above	90.60	NIH 5 or less	34 days
H. Fan; 2021	Composite (ischemic stroke, TIA, MI, & moderate to severe bleeding events).	Moderate-to-severe bleeding defined by the GUSTO criteria.	61 (IQR 53-68)	Not specified	72.50	0.0	Not applicable	100	NIHSS 5 and less	Not so clear, appears to be between 21 and 30 days.

Y. Gao; INSPIRES; 2023	New stroke (ischemic or hemorrhagic) within 90 days.	Moderate-to-severe bleeding (GUSTO definition)	65	35 to 80 years	64.20	13.1	4 and above	86.90	NIHSS 5 and less	90 days
RA. Algarni; 2023	Composite (recurrent ischemic stroke, rehospitalization & all-cause mortality) at the end of the 59-month follow-up period.	-	62.5	Above 18	65.20	They were excluded	Not excluded	100.00	Not mentioned	59 months
L. Wang; 2023	Recurrent stroke at 1 year	-	62.4	Not specified	68.00	0.0	Not applicable	100	NIHSS 4 -10	12 months
T. Deng; 2023	Recurrent ischemic stroke	-	65	18 and above	72.10	0.0	Not applicable	100	NIHSS less than 10	90 days
T. Liu; SEACOAST; 2024	Composite (ischemic stroke recurrence, TIA, symptomatic intracerebral hemorrhage, MI or angina attacks & vascular death)	Severe bleeding (GUSTO criteria)	61.7	Not specified	73.30	0.0	Not applicable	100	NIHSS 5 and less	90 days

VR. Suryawanshi; 2025	Composite (early neurological deterioration and new stroke or TIA.	-	59.2	Not specified	62.00	12.0	4 and above	88	NIHSS 10 and less	3 months

Abbreviations: DAPT- dual antiplatelet therapy; GUSTO- Global Use of Strategies to Open Occluded Arteries; MI- myocardial infarction; mRS- modified Rankin Scale; NIHSS- National Institutes of Health Stroke Scale; RIND- reversible ischemic neurologic deficit; SAPT- single antiplatelet therapy; TIA- transient ischemic attack.

Supplementary Table S2. **High-visibility studies excluded from the meta-analysis and reasons for exclusion.**

Study (Year)	Author	Population / Design	Reason for Exclusion	Comment
CARESS (2005)	Hugh S Markus, et al	Carotid stenosis; SAPT vs DAPT; outcome = microembolic signals	Wrong population and wrong outcome	Mechanistic, surrogate imaging endpoint
CLAIR (2010)	Ka Sing Lawrence Wong, et al	Large-artery stenosis; outcome = microembolic signals	Wrong population and wrong outcome	Mechanistic, surrogate microembolic signal endpoint
SPS3 (2012)	The SPS3 Investigators	Patients with lacunar (small-vessel) stroke only	Wrong population	Subtype-specific (small-vessel disease) not generalizable to unselected TIA/ischemic stroke
COMPRESS (2016)	Keun-Sik Hong, et al	Intracranial atherosclerotic stenosis	Wrong population	Targeted stenotic disease subgroup
CATHARSIS (2015)	Shinichiro Uchiyama, et al	Intracranial atherosclerosis; cilostazol + ASA vs ASA	Wrong population and surrogate outcome	Imaging endpoint; not clinical recurrent stroke
TARDIS (2018)	Prof Philip M Bath, et al	DAPT vs triple therapy (aspirin + clopidogrel + dipyridamole)	Wrong intervention	Not comparable to DAPT vs SAPT
READAPT (2024)	Eleonora De Matteis , et al	Real-life study on short-term Dual Antiplatelet treatment in Patients with ischemic stroke or Transient ischemic attack	Wrong population	Patients with even severe strokes were included. No stringent NIHSS and $ABCD^2$ score cut-offs were applied

Footnotes: SAPT = single antiplatelet therapy; DAPT = dual antiplatelet therapy; ASA = aspirin.

Supplementary Table S3: **Cochrane Risk of Bias 2 summary for randomized trials included in the metanalysis.**

Study	Randomization Process	Deviations from Intended Interventions	Missing Outcome Data	Measurement of Outcome	Selective Reporting	Overall Bias
American-Canadian Study Group	✅	✅	✅	✅	✅	✅
J. Matías-Guiu, et al.	✅	✅	✅	✅	✅	✅
H-C. Diener, et al; ESPS 2 Trial	✅	✅	✅	✅	✅	✅
H-C. Diener, et al; MATCH trial	✅	✅	✅	✅	✅	✅
ESPRIT Study Group	✅	✅	✅	✅	✅	✅
J. Kennedy, et al; FASTER trial	✅	⚠️	✅	✅	✅	⚠️
RL. Sacco, et al; PRoFESS trial	✅	⚠️	✅	✅	✅	⚠️
S. Uchiyama, et al; JASAP trial	✅	✅	✅	✅	✅	✅
T. Nakamura, et al.	✅	✅	✅	✅	✅	✅
Y. Wang, et al; CHANCE trial	✅	✅	✅	✅	✅	✅
F. He et al	✅	✅	✅	⚠️	✅	⚠️
SC. Johnston, et	✅	✅	✅	✅	✅	✅

al; POINT trial						
J. Aoki, et al; ADS trial	✅	✅	✅	✅	✅	✅
K. Toyoda, et al; CSPS.com Trial	✅	✅	✅	✅	✅	✅
M. Khazaei, et al	✅	✅	✅	✅	✅	✅
S.C. Johnston, et al; THALES re-analysis	✅	✅	✅	✅	✅	✅
Y. Gao, et al; INSPIRES trial	✅	✅	✅	✅	✅	✅
T. Deng, et al.	✅	✅	✅	✅	⚠️	⚠️

✅ - low risk of bias, ⚠️ - some concerns for bias

Supplementary Table S4: **Newcastle-Ottawa Scale (NOS) Star Summary with Visual Overall Quality for the cohort studies that were included in the metanalysis.**

Study	Selection (4)	Comparability (2)	Outcome/Exposure (3)	Total NOS (9)	Overall Quality
Y. Wang, et al; CHANCE long-term follow-up	⭐⭐⭐⭐	⭐⭐	⭐⭐⭐	9/9	✅ High
CB. Christiansen, et al.	⭐⭐⭐⭐	⭐⭐	⭐⭐⭐	9/9	✅ High
J-T Kim et al.	⭐⭐⭐⭐	⭐⭐	⭐⭐	8/9	⚠️ Moderate
H-L. Lee, et al.	⭐⭐⭐	⭐⭐	⭐⭐⭐	8/9	⚠️ Moderate
H. Fan, et al.	⭐⭐⭐	⭐	⭐⭐⭐	7/9	⚠️ Moderate
RA. Algarni, et al.	⭐⭐⭐	⭐	⭐⭐⭐	7/9	⚠️ Moderate
L. Wang, et al.	⭐⭐⭐	⭐⭐	⭐⭐⭐	8/9	⚠️ Moderate
T. Liu et al.	⭐⭐⭐⭐	⭐⭐	⭐⭐⭐	9/9	✅ High
VR. Suryawanshi, et al.	⭐⭐⭐	⭐⭐	⭐⭐⭐	8/9	⚠️ Moderate

Supplementary Table S5: **GRADE summary of certainty of evidence for the main outcomes in the meta-analysis comparing dual antiplatelet therapy versus single antiplatelet therapy in patients with transient ischemic attacks or ischemic stroke.**

Outcome	No. of Studies	Participants	Risk of Bias	Inconsistency	Indirectness	Imprecision	Publication Bias	Overall Certainty
**Stroke recurrence**	27	123,136	Not serious	Serious (I^2 = 57.0%)	Not serious	Not serious	Not serious	High
**Major bleeding**	15	77,517	Serious	Serious (I^2=60.4%)	Not serious	Not Serious	None suspected	Moderate

Footnotes:

1. Stroke recurrence was downgraded one level for inconsistency due to moderate heterogeneity (I^2 = 57%) and potential small-study effects; overall rated as *high certainty* based on the predominance of high-quality RCTs and consistency of direction across subgroups.

2 Major bleeding was downgraded for serious risk of bias (some concerns in a few RCTs per RoB 2.0), inconsistency (I^2 = 60%), and moderate imprecision (confidence interval borders unity); overall rated as *moderate certainty*.

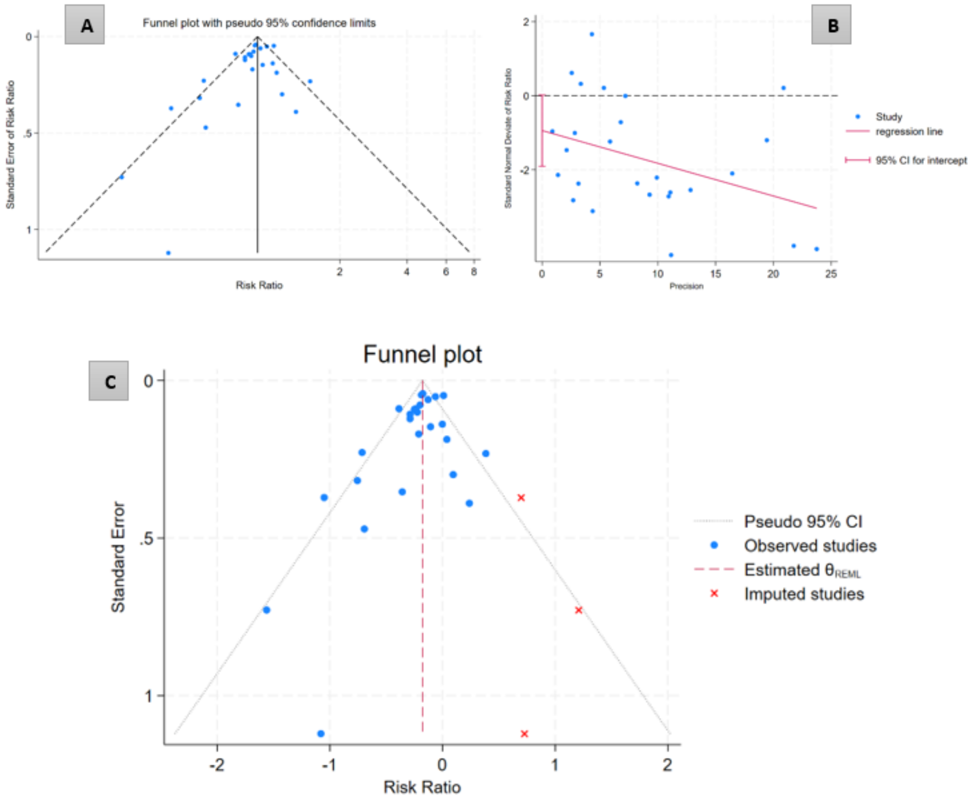

Supplementary Figure S1. **Assessment of publication bias for the primary efficacy outcome (recurrent stroke). Funnel plot (Panel A) and Egger's regression plot (Panel B) indicate possible small-study effects. Trim-and-fill analysis (Panel C) identified three potentially missing studies, with minimal change in the pooled effect estimate, supporting the robustness of the results**.

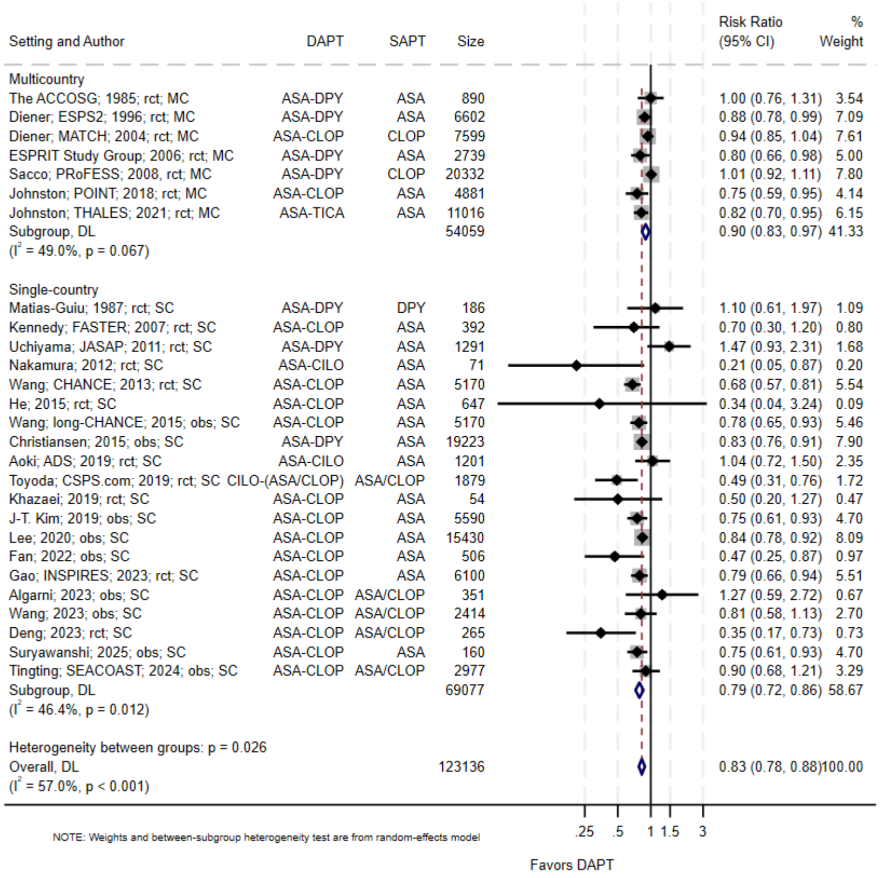

Supplementary Figure S2. **Subgroup analysis of the efficacy outcome (recurrent stroke prevention) by geographical setting of the included studies.**

Abbreviations: ASA-aspirin; CLOP-clopidogrel; CILO-cilostazol; DAPT-dual antiplatelet therapy; DPY-dipyridamole; MC-multicountry; obs-observational cohort; rct-randomized trial; SC-single country; SAPT-single antiplatelet therapy. **Footnote:** Study details are presented as the first author; ± study acronym; publication year; study design; study setting. *ASA–CLOP* denotes the aspirin–clopidogrel combination, and *ASA/CLOP* indicates either aspirin or clopidogrel monotherapy. The same notation applies to other dual and single antiplatelet combinations.

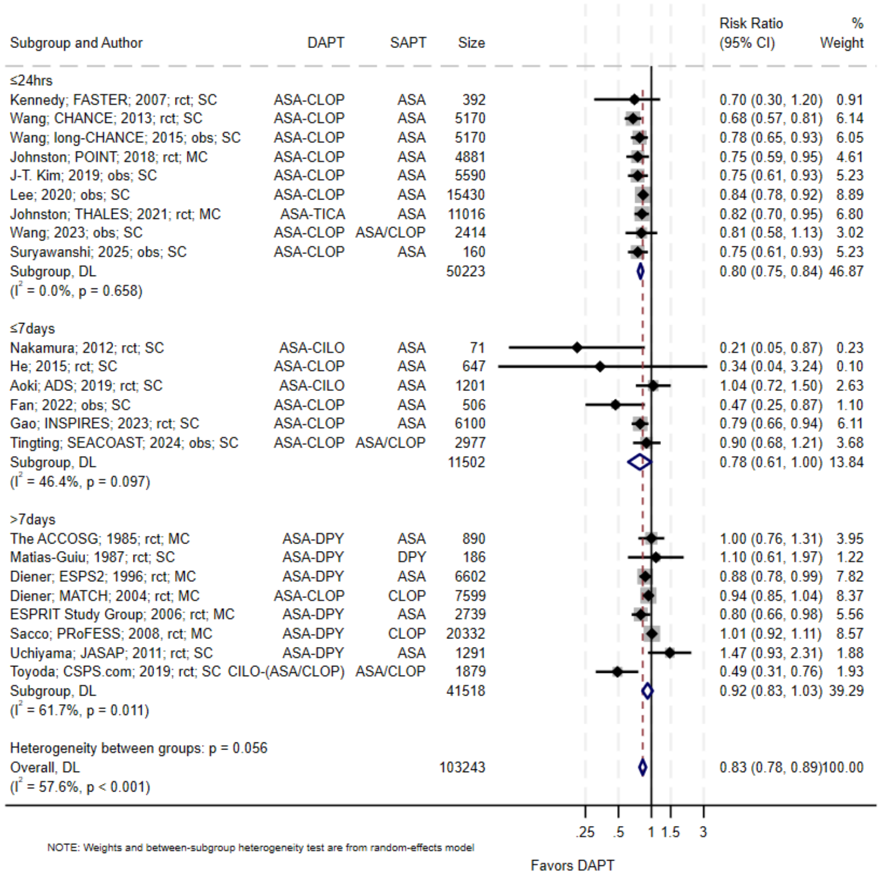

Supplementary Figure S3. **Subgroup analysis of the efficacy outcome (recurrent stroke prevention) by time from symptom onset to dual antiplatelet therapy initiation.**

Abbreviations: ASA-aspirin; CLOP-clopidogrel; CILO-cilostazol; DAPT-dual antiplatelet therapy; DPY-dipyridamole; MC-multicountry; obs-observational cohort; rct-randomized trial; SC-single country; SAPT-single antiplatelet therapy. **Footnote:** Study details are presented as the first author; ± study acronym; publication year; study design; study setting. *ASA–CLOP* denotes the aspirin–clopidogrel combination, and *ASA/CLOP* indicates either aspirin or clopidogrel monotherapy. The same notation applies to other dual and single antiplatelet combinations.

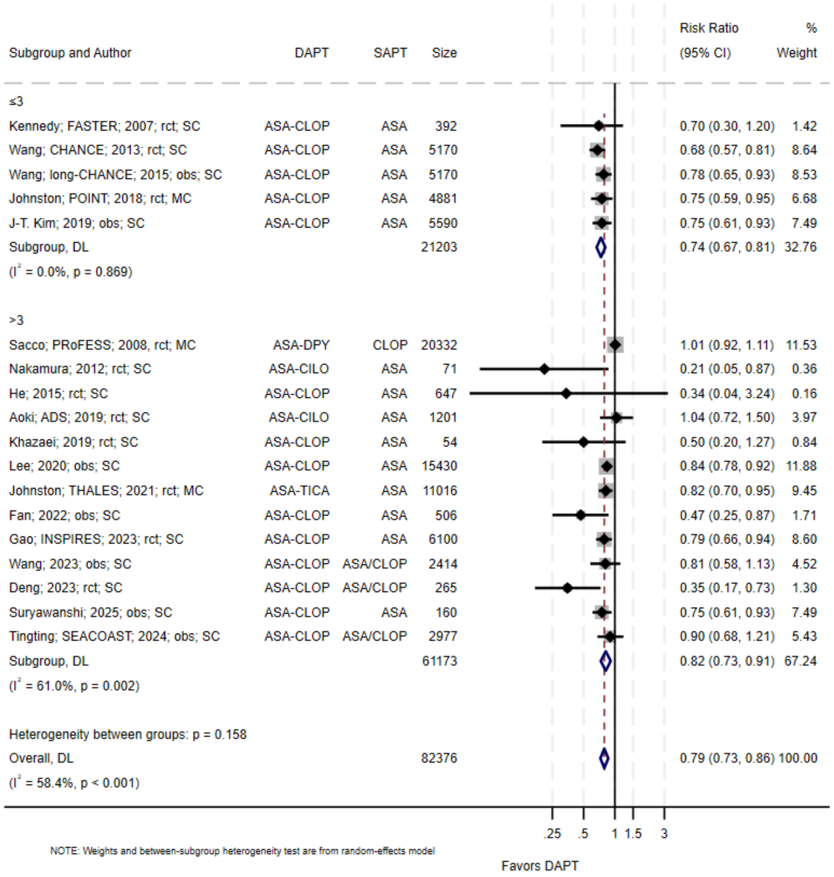

Supplementary Figure S4. **Subgroup analysis of the efficacy outcome (recurrent stroke prevention) by baseline National Institute of Health Stroke Scale (NIHSS).**

Abbreviations: ASA-aspirin; CLOP-clopidogrel; CILO-cilostazol; DAPT-dual antiplatelet therapy; DPY-dipyridamole; MC-multicountry; obs-observational cohort; rct-randomized trial; SC-single country; SAPT-single antiplatelet therapy. **Footnote:** Study details are presented as the first author; ± study acronym; publication year; study design; study setting. *ASA–CLOP* denotes the aspirin–clopidogrel combination, and *ASA/CLOP* indicates either aspirin or clopidogrel monotherapy. The same notation applies to other dual and single antiplatelet combinations.

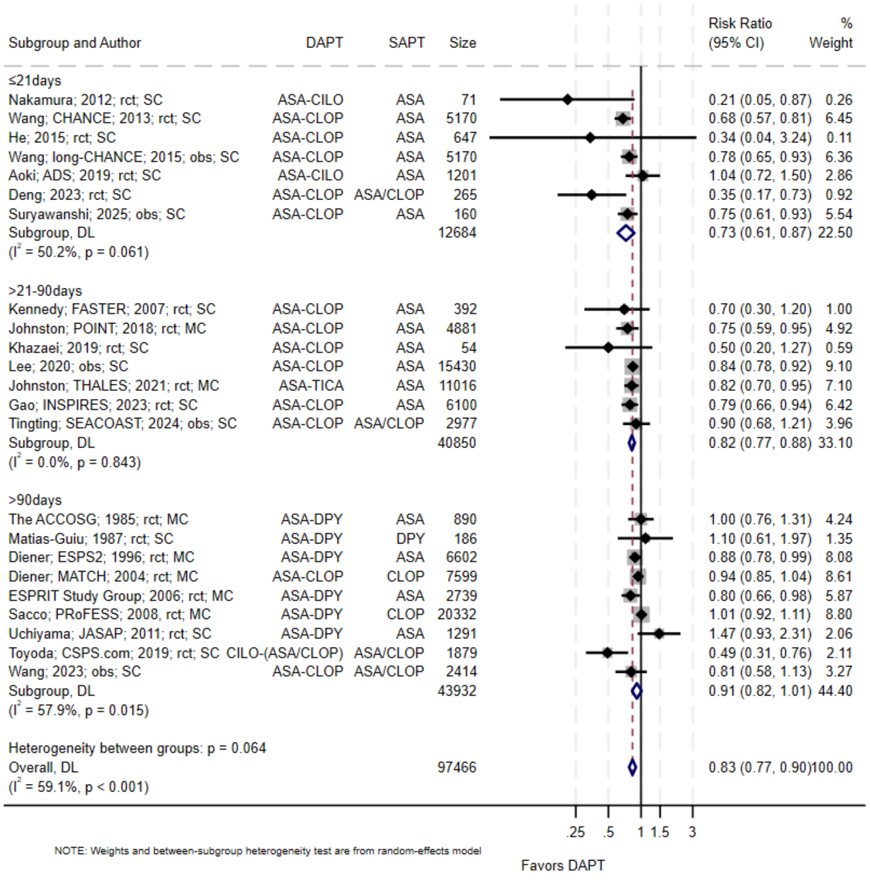

Supplementary Figure S5. **Subgroup analysis of the efficacy outcome (recurrent stroke prevention) by duration of dual antiplatelet therapy.**

Abbreviations: ASA-aspirin; CLOP-clopidogrel; CILO-cilostazol; DAPT-dual antiplatelet therapy; DPY-dipyridamole; MC-multicountry; obs-observational cohort; rct-randomized trial; SC-single country; SAPT-single antiplatelet therapy. **Footnote:** Study details are presented as the first author; ± study acronym; publication year; study design; study setting. *ASA–CLOP* denotes the aspirin–clopidogrel combination, and *ASA/CLOP* indicates either aspirin or clopidogrel monotherapy. The same notation applies to other dual and single antiplatelet combinations.

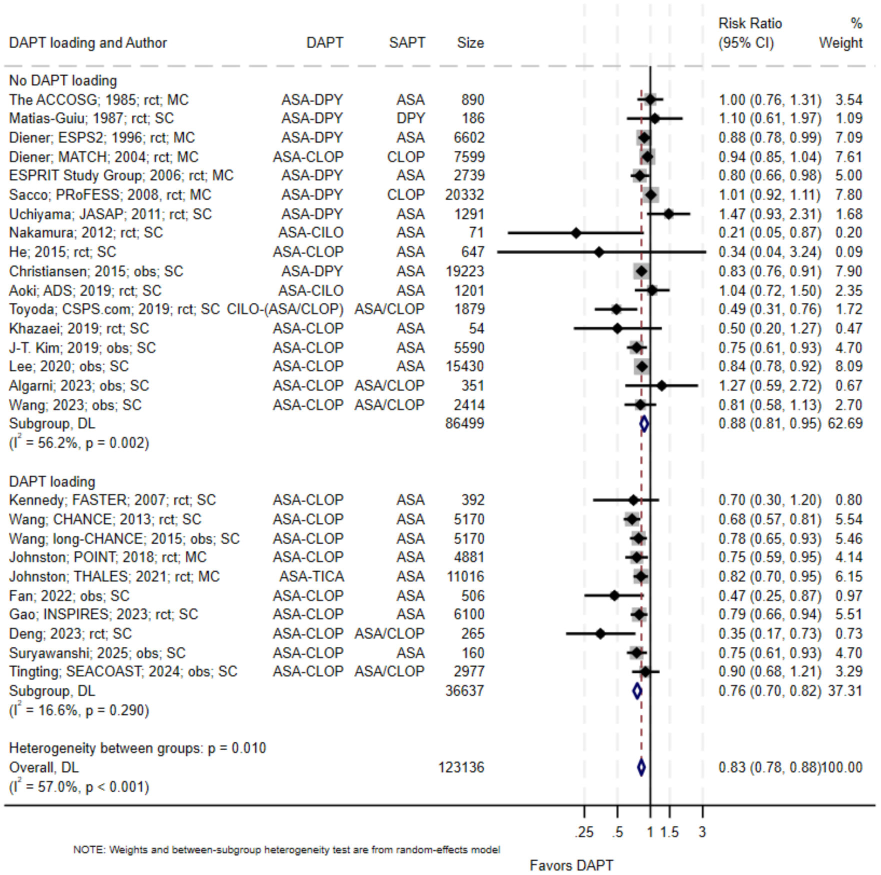

Supplementary Figure S6. **Subgroup analysis of the efficacy outcome (recurrent stroke prevention) by use of an initial loading-dose strategy.**

Abbreviations: ASA-aspirin; CLOP-clopidogrel; CILO-cilostazol; DAPT-dual antiplatelet therapy; DPY-dipyridamole; MC-multicountry; obs-observational cohort; rct-randomized trial; SC-single country; SAPT-single antiplatelet therapy. **Footnote:** Study details are presented as the first author; ± study acronym; publication year; study design; study setting. *ASA–CLOP* denotes the aspirin–clopidogrel combination, and *ASA/CLOP* indicates either aspirin or clopidogrel monotherapy. The same notation applies to other dual and single antiplatelet combinations.

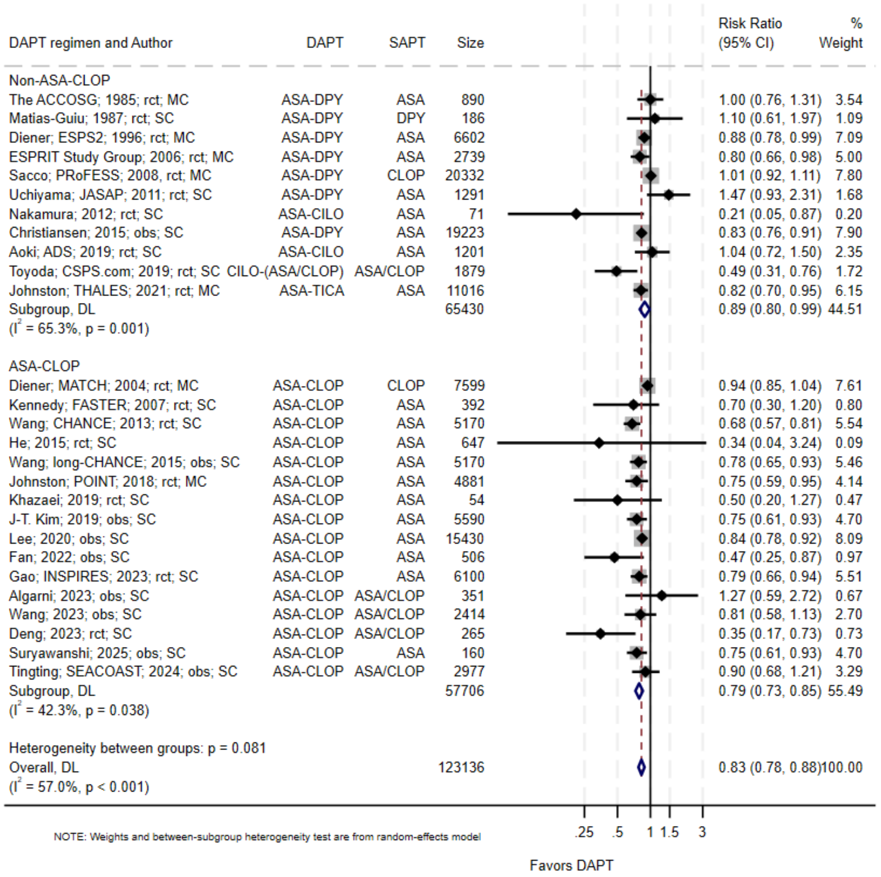

Supplementary Figure S7. **Subgroup analysis of the efficacy outcome (recurrent stroke prevention) by dual antiplatelet combination used.**

Abbreviations: ASA-aspirin; CLOP-clopidogrel; CILO-cilostazol; DAPT-dual antiplatelet therapy; DPY-dipyridamole; MC-multicountry; obs-observational cohort; rct-randomized trial; SC-single country; SAPT-single antiplatelet therapy. **Footnote:** Study details are presented as the first author; ± study acronym; publication year; study design; study setting. *ASA–CLOP* denotes the aspirin–clopidogrel combination, and *ASA/CLOP* indicates either aspirin or clopidogrel monotherapy. The same notation applies to other dual and single antiplatelet combinations. *Non–ASA–CLOP denotes other dual antiplatelet combinations beyond aspirin–clopidogrel, such as aspirin–ticagrelor, aspirin–cilostazol, aspirin–dipyridamole, or cilostazol–clopidogrel.*

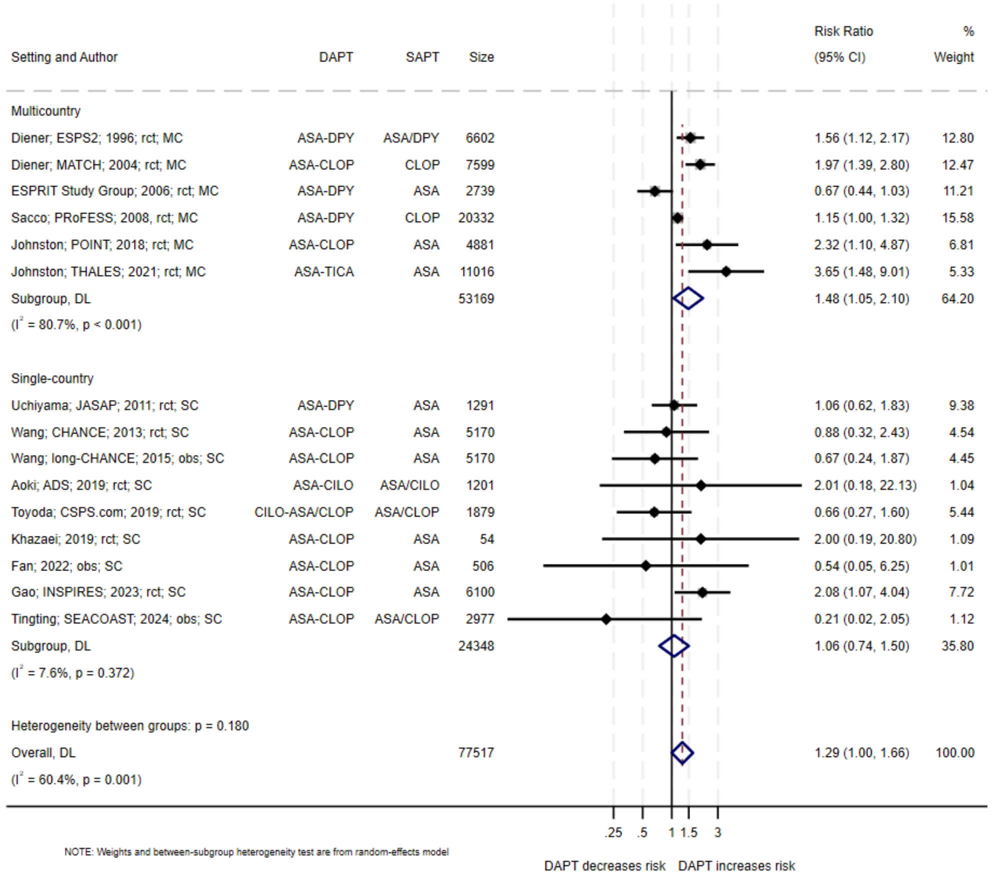

Supplementary Figure S8. **Subgroup analysis of the safety outcome (major bleeding) by geographical setting of the included studies.**

Abbreviations: ASA-aspirin; CLOP-clopidogrel; CILO-cilostazol; DAPT-dual antiplatelet therapy; DPY-dipyridamole; MC-multicountry; obs-observational cohort; rct-randomized trial; SC-single country; SAPT-single antiplatelet therapy. **Footnote:** Study details are presented as the first author; ± study acronym; publication year; study design; study setting. *ASA–CLOP* denotes the aspirin–clopidogrel combination, and *ASA/CLOP* indicates either aspirin or clopidogrel monotherapy. The same notation applies to other dual and single antiplatelet combinations.

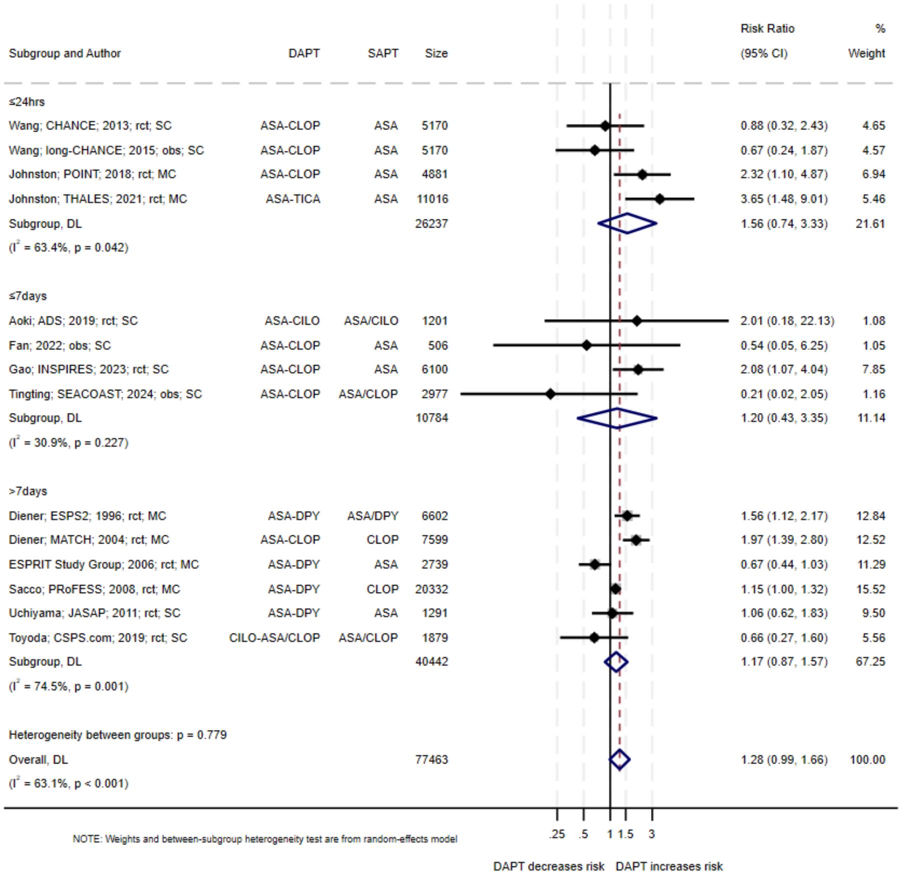

Supplementary Figure S9. **Subgroup analysis of the safety outcome (major bleeding) by time from symptom onset to dual antiplatelet therapy initiation.**

Abbreviations: ASA-aspirin; CLOP-clopidogrel; CILO-cilostazol; DAPT-dual antiplatelet therapy; DPY-dipyridamole; MC-multicountry; obs-observational cohort; rct-randomized trial; SC-single country; SAPT-single antiplatelet therapy. **Footnote:** Study details are presented as the first author; ± study acronym; publication year; study design; study setting. *ASA–CLOP* denotes the aspirin–clopidogrel combination, and *ASA/CLOP* indicates either aspirin or clopidogrel monotherapy. The same notation applies to other dual and single antiplatelet combinations.

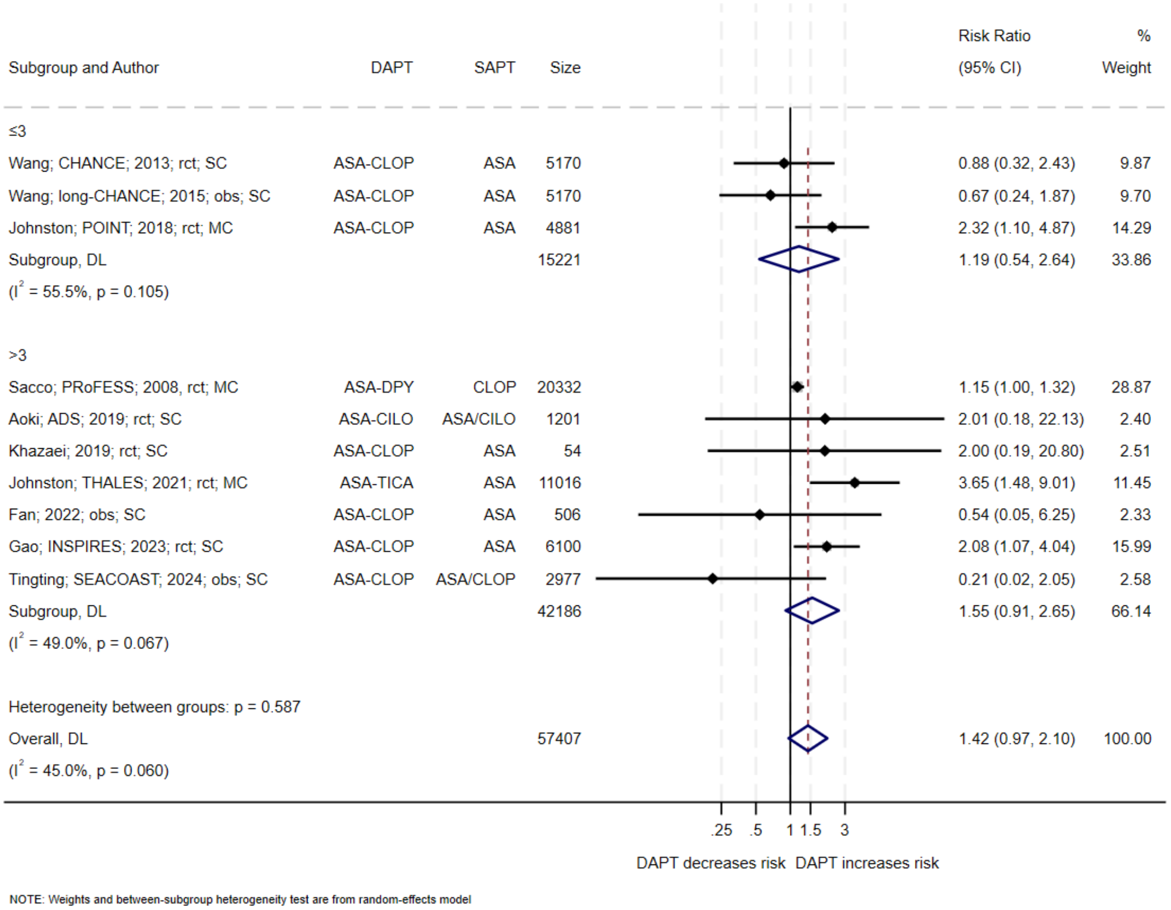

Supplementary Figure S10. **Subgroup analysis of the safety outcome (major bleeding) by baseline National Institute of Health Stroke Scale (NIHSS).**

Abbreviations: ASA-aspirin; CLOP-clopidogrel; CILO-cilostazol; DAPT-dual antiplatelet therapy; DPY-dipyridamole; MC-multicountry; obs-observational cohort; rct-randomized trial; SC-single country; SAPT-single antiplatelet therapy. **Footnote:** Study details are presented as the first author; ± study acronym; publication year; study design; study setting. *ASA–CLOP* denotes the aspirin–clopidogrel combination, and *ASA/CLOP* indicates either aspirin or clopidogrel monotherapy. The same notation applies to other dual and single antiplatelet combinations.

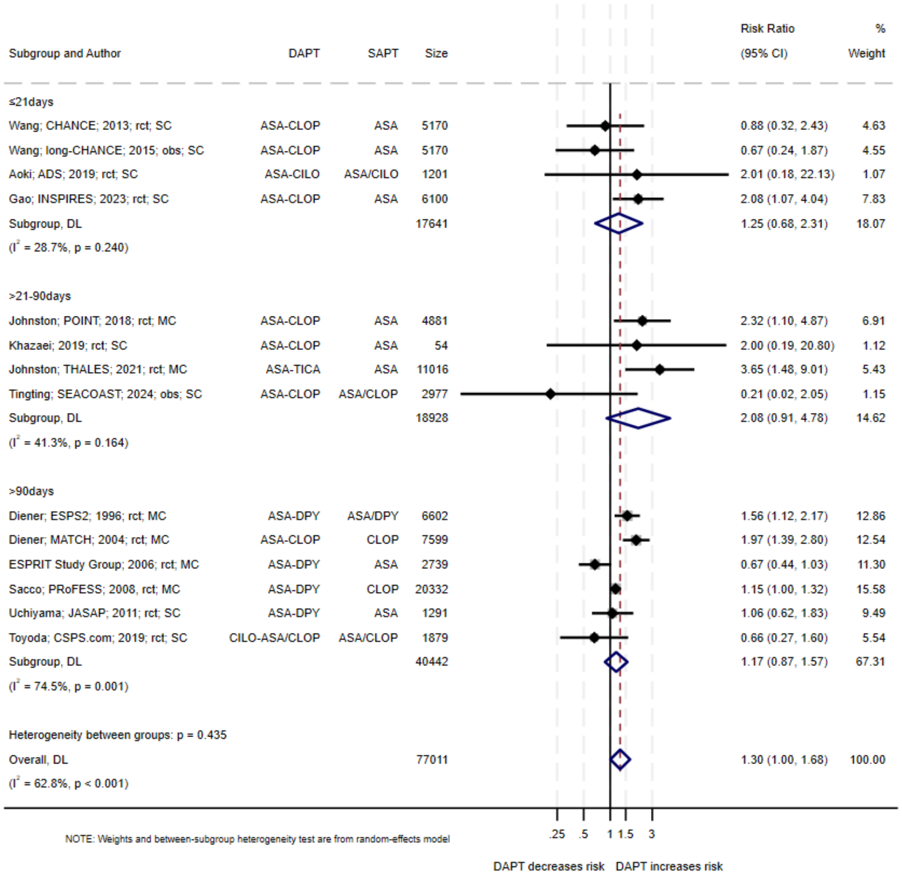

Supplementary Figure S11. **Subgroup analysis of the safety outcome (major bleeding) by duration of dual antiplatelet therapy.**

Abbreviations: ASA-aspirin; CLOP-clopidogrel; CILO-cilostazol; DAPT-dual antiplatelet therapy; DPY-dipyridamole; MC-multicountry; obs-observational cohort; rct-randomized trial; SC-single country; SAPT-single antiplatelet therapy. **Footnote:** Study details are presented as the first author; ± study acronym; publication year; study design; study setting. *ASA–CLOP* denotes the aspirin–clopidogrel combination, and *ASA/CLOP* indicates either aspirin or clopidogrel monotherapy. The same notation applies to other dual and single antiplatelet combinations.

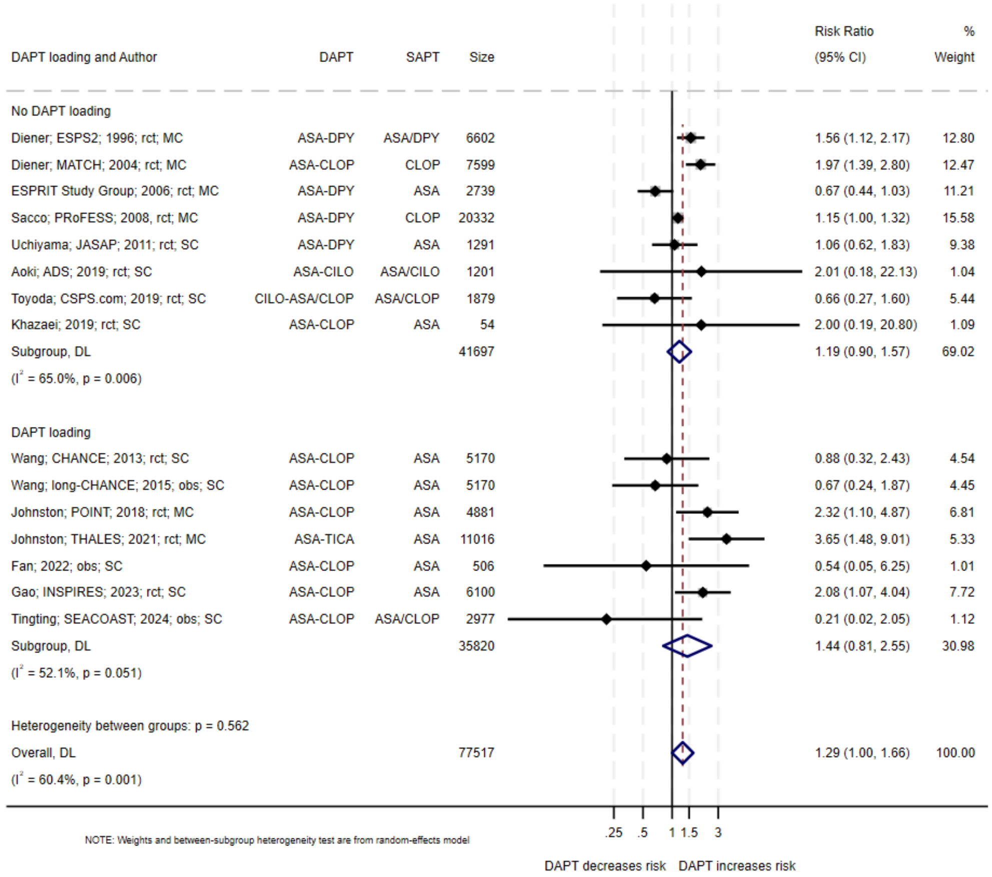

Supplementary Figure S12. **Subgroup analysis of the safety outcome (major bleeding) by use of an initial loading-dose strategy.**

Abbreviations: ASA-aspirin; CLOP-clopidogrel; CILO-cilostazol; DAPT-dual antiplatelet therapy; DPY-dipyridamole; MC-multicountry; obs-observational cohort; rct-randomized trial; SC-single country; SAPT-single antiplatelet therapy. **Footnote:** Study details are presented as the first author; ± study acronym; publication year; study design; study setting. *ASA–CLOP* denotes the aspirin–clopidogrel combination, and *ASA/CLOP* indicates either aspirin or clopidogrel monotherapy. The same notation applies to other dual and single antiplatelet combinations.

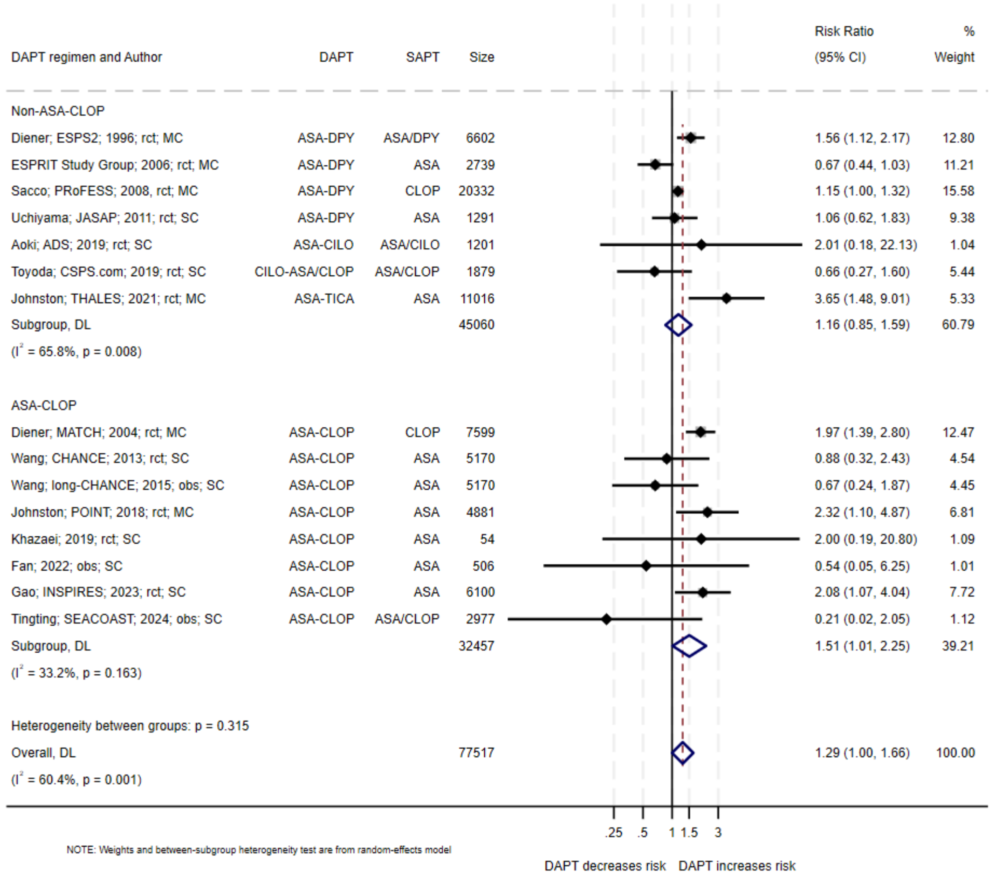

Supplementary Figure S13. **Subgroup analysis of the safety outcome (major bleeding) by dual antiplatelet combination used.**

Abbreviations: ASA-aspirin; CLOP-clopidogrel; CILO-cilostazol; DAPT-dual antiplatelet therapy; DPY-dipyridamole; MC-multicountry; obs-observational cohort; rct-randomized trial; SC-single country; SAPT-single antiplatelet therapy. **Footnote:** Study details are presented as the first author; ± study acronym; publication year; study design; study setting. *ASA–CLOP* denotes the aspirin–clopidogrel combination, and *ASA/CLOP* indicates either aspirin or clopidogrel monotherapy. The same notation applies to other dual and single antiplatelet combinations. *Non–ASA–CLOP denotes other dual antiplatelet combinations beyond aspirin–clopidogrel, such as aspirin–ticagrelor, aspirin–cilostazol, aspirin–dipyridamole, or cilostazol–clopidogrel.*

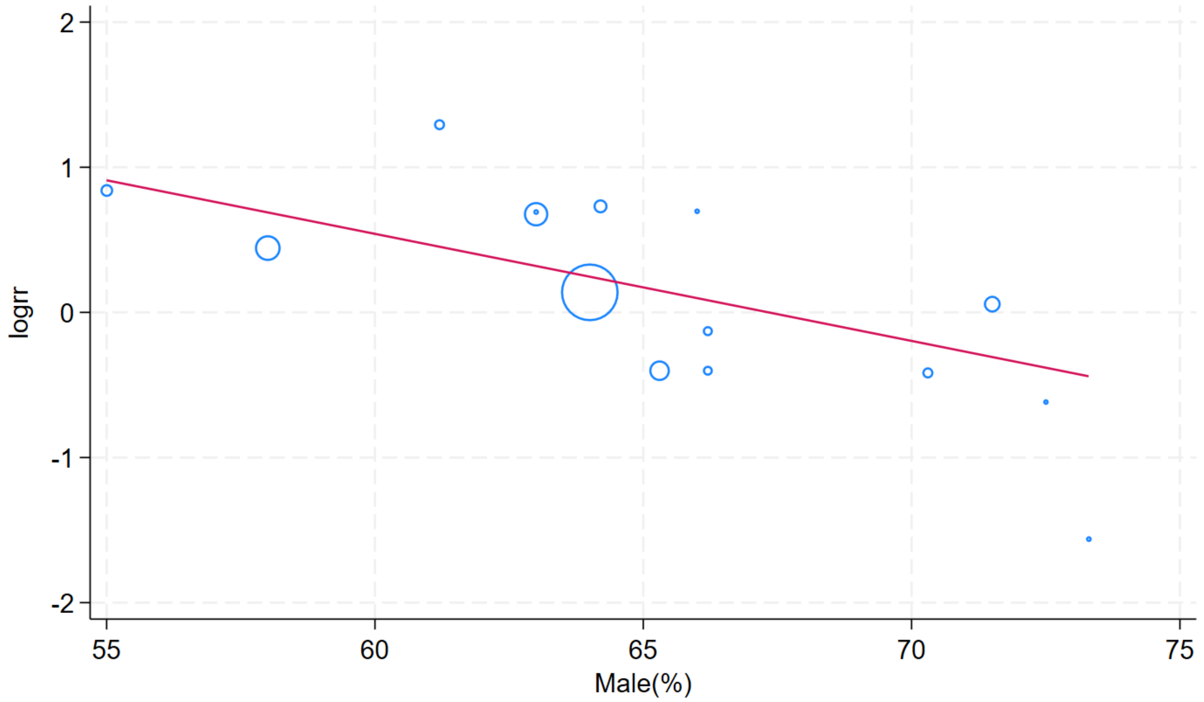

Supplementary Figure S14**. Meta-regression analysis evaluating the association between the proportion of male participants and major bleeding risk comparing dual antiplatelet therapy (DAPT) versus single antiplatelet therapy (SAPT).** Each bubble represents a study, with bubble size proportional to study weight. The regression line (β = –0.07; p = 0.02) indicates an inverse relationship between male proportion and bleeding risk, suggesting relatively higher susceptibility to DAPT-related bleeding among female participants.
