# Supplementary material for: Dual antiplatelet therapy use after non-cardioembolic ischemic stroke or transient ischemic attack: a meta-analysis of trials and cohort studies
Source: Front Neurol. 2026 Jan 12;16:1750241. doi: 10.3389/fneur.2025.1750241 (PMC12869719; doi:10.3389/fneur.2025.1750241)
Supplement: Supplementary file 2 [file Data_Sheet_2.pdf]

PRISMA 2020 for Abstracts checklist.

Section and Topic	Item #	Checklist item	Reported (Yes/No)
**TITLE**			
Title	1	Identify the report as a systematic review.	Yes
**BACKGROUND**			
Objectives	2	Provide an explicit statement of the main objective(s) or question(s) the review addresses.	Yes
**METHODS**			
Eligibility criteria	3	Specify the inclusion and exclusion criteria for the review.	Yes
Information sources	4	Specify the information sources (e.g. databases, registers) used to identify studies and the date when each was last searched.	Yes
Risk of bias	5	Specify the methods used to assess risk of bias in the included studies.	Yes
Synthesis of results	6	Specify the methods used to present and synthesise results.	Yes
**RESULTS**			
Included studies	7	Give the total number of included studies and participants and summarise relevant characteristics of studies.	Yes
Synthesis of results	8	Present results for main outcomes, preferably indicating the number of included studies and participants for each. If meta-analysis was done, report the summary estimate and confidence/credible interval. If comparing groups, indicate the direction of the effect (i.e. which group is favoured).	Yes
**DISCUSSION**			
Limitations of evidence	9	Provide a brief summary of the limitations of the evidence included in the review (e.g. study risk of bias, inconsistency and imprecision).	Yes
Interpretation	10	Provide a general interpretation of the results and important implications.	Yes
**OTHER**			
Funding	11	Specify the primary source of funding for the review.	Yes
Registration	12	Provide the register name and registration number.	Yes

PRISMA 2020 Checklist for DAPT vs SAPT Systematic Review.

Section & Topic	Item	Checklist item	Location in manuscript (section)
**TITLE**	1	Identify the report as a systematic review.	Dual Antiplatelet Use After Ischemic Stroke or TIA: A Meta-analysis of Trials and Cohort Studies.
**ABSTRACT**	2	See PRISMA 2020 for Abstracts checklist.	Abstract section
**INTRODUCTION**	3	Rationale: Describe the rationale for the review.	Introduction, 1st-4th paragraphs
	4	Objectives: Provide an explicit statement of the objectives.	Last paragraph of Introduction
**METHODS**	5	Eligibility criteria: Inclusion and exclusion criteria, and how studies were grouped.	Methods: Eligibility Criteria and Grouping for Synthesis sections
	6	Information sources: Databases and date last searched.	Methods: Search Strategy
	7	Search strategy: Full search strategies for all databases.	In the protocol registered with PROSPERO (CRD420251017979)
	8	Selection process: How studies were selected.	Methods: Study Selection
	9	Data collection process: How data were collected.	Methods: Data Extraction & Methodological Quality Assessment, and Data Synthesis
	10a	Data items: List and define outcomes sought.	Methods: Eligibility Criteria (PICOS criteria)
	10b	Other variables collected (e.g., patient characteristics).	Methods: Data Synthesis
	11	Risk of bias assessment.	Methods: Data Extraction & Methodological Quality Assessment
	12	Effect measures used.	Methods: Eligibility Criteria (PICOS)

	13a	Processes to decide study eligibility for each synthesis.	Methods: Search Strategy & Study Selection
	13b	Data preparation (e.g., conversions).	Methods: Data Synthesis
	13c	Methods to tabulate/display results.	Results: Tables and Figures
	13d	Synthesis methods, including heterogeneity measures.	Methods: Data Synthesis
	13e	Methods to explore causes of heterogeneity (subgroup analyses).	Methods: Data Synthesis
	13f	Sensitivity analyses.	Methods: Data Synthesis
	14	Risk of bias due to missing results (publication bias).	Methods: Data Synthesis, Results: Funnel plots, Egger's test
	15	Certainty assessment.	Methods: Certainty of Evidence
**RESULTS**	16a	Search and selection results (with flow diagram).	Results: Study Selection, Figure 1 (PRISMA diagram)
	16b	Studies excluded at full-text stage and reasons.	Figure 1 (PRISMA diagram)
	17	Characteristics of included studies.	Results: Study Characteristics (Table 1) and Supplementary Table S1.
	18	Risk of bias in included studies.	**Results:** QUALITY ASSESSMENT FOR INCLUDED STUDIES**,** Supplementary Tables S3 & S4
	19	Results of individual studies.	Results: Figures 2 & 4
	20a	Characteristics and risk of bias of contributing studies.	Results: QUALITY ASSESSMENT FOR INCLUDED STUDIES**,** Supplementary Tables S3 & S4
	20b	Statistical syntheses (meta-analyses): summary estimate, precision, heterogeneity.	Results: Efficacy and safety outcomes, Figures 2& 4, Supplementary Data

	20c	Causes of heterogeneity.	Results: Subgroup analyses, Figure 3, Supplementary data
	20d	Sensitivity analyses.	Results: Funnel plots, Egger's test, trim-and-fill
	21	Reporting bias risk assessments.	Results: QUALITY ASSESSMENT FOR INCLUDED STUDIES**,** Supplementary Tables S3 & S4
	22	Certainty of evidence.	Results: CERTAINTY OF EVIDENCE, GRADE system used
**DISCUSSION**	23a	Interpretation of results in context.	Discussion
	23b	Limitations of the evidence.	Certainty of Evidence and Study Limitations.
	23c	Limitations of the review process.	Study Limitations
	23d	Implications for practice, policy, future research.	Clinical Implications and Future Directions
**OTHER INFORMATION**	24a	Registration: Provide register name and registration number.	Methods: PROSPERO registration
	24b	Protocol access or state that no protocol was prepared.	Methods: PROSPERO registration
	24c	Amendments to information in protocol.	Not explicitly stated- no major amendments.
	25	Support: Funding and role of funders.	Methods: This review received no funding
	26	Competing interests.	Title page: Conflict of Interest Statement ("The authors have no conflicts of interest.")
	27	Availability of data, code, and materials.	Supplementary information
